# A Theoretical Exploration of the Photoinduced Breaking Mechanism of the Glycosidic Bond in Thymine Nucleotide

**DOI:** 10.3390/molecules29163789

**Published:** 2024-08-10

**Authors:** Xiao Huang, Yuuichi Orimoto, Yuriko Aoki

**Affiliations:** 1 Department of Interdisciplinary Engineering Sciences, Chemistry and Materials Science, Interdisciplinary Graduate School of Engineering Sciences, Kyushu University, 6-1 Kasuga Park, Fukuoka 816-8580, Japan; huang.xiao.932@s.kyushu-u.ac.jp; 2 Department of Material Sciences, Faculty of Engineering Sciences, Kyushu University, 6-1 Kasuga Park, Fukuoka 816-8580, Japan; orimoto.yuuichi.888@m.kyushu-u.ac.jp

**Keywords:** DNA, glycosidic bond cleavage, DFT/TDDFT

## Abstract

DNA glycosidic bond cleavage may induce cancer under the ultraviolet (UV) effect. Yet, the mechanism of glycosidic bond cleavage remains unclear and requires more detailed clarification. Herein, quantum chemical studies on its photoinduced mechanism are performed using a 5′-thymidine monophosphate (5′-dTMPH) model. In this study, four possible paths were examined to study the glycosidic bond cleavage. The results showed that, upon excitation, the electronic transition from the π bonding to π antibonding orbitals of the thymine ring leads to the damage of the thymine ring. Afterwards, the glycosidic bond is cleaved. At first, the doublet ground state (GS) path of glycosidic bond cleavage widely studied by other groups is caused by free electron generated by photoirradiation, with a kinetically feasible energy barrier of ~23 kcal/mol. Additionally, then, the other three paths were proposed that also might cause the glycosidic bond cleavage. The first one is the doublet excited state (ES) path, triggered by free electron along with UV excitation, which can result in a very-high-energy barrier ~49 kcal/mol that is kinetically unfavorable. The second one is the singlet ES path, induced by direct UV excitation, which assumes DNA is directly excited by UV light, which features a very low-energy barrier ~16 kcal/mol that is favored in kinetics. The third one is the triplet ES path, from the singlet state via intersystem crossing (ISC), which refers to a feasible ~27 kcal/mol energy barrier. This study emphasizes the pivotal role of the DNA glycosidic bond cleavage by our proposed direct UV excitation (especially singlet ES path) in addition to the authorized indirect free-electron-induced path, which should provide essential insights to future mechanistic comprehension and novel anti-cancer drug design.

## 1. Introduction

The interaction between UV radiation and cellular DNA can lead to DNA damage, such as DNA strand damage, sugar destruction and base dimer formation, etc., further resulting in the formation of cancer [1,2,3,4,5,6,7,8,9,10,11]. DNA itself can randomly interact with the primary high-energy particles produced by UV radiation, resulting in the ionization (forming DNA radical cation and secondary electrons), excitation (DNA*) and reduction (DNA reacting with secondary electrons generated from ionization to form DNA radical anion) [4,5,8]. When the ejected secondary electrons (an estimated quantity of ~4 × 10^4^/MeV) [12] lose energy, low-energy electrons are generated [3]. Once a low-energy electron is captured by a DNA molecule, the excess energy of low-energy electrons can be transferred to the DNA, leading to the formation of a transient negative ion, finally cleaving the DNA chemical bond [1,2,3,6,7,8]. Low-energy electrons are more effective at triggering DNA bond scission due to their tendency for interaction with DNA molecules and ability to transfer enough energy to cleave DNA bonds, while high-energy electrons from UV radiation tend to cause direct ionization [1,2,3,4,5,6,7,8]. Therefore, DNA damage induced by low-energy electrons has attracted extensive attention [10,11,12,13,14,15,16,17,18,19,20,21,22,23,24], in which a crucial route is through the glycosidic bond cleavage under UV radiation. The experimental [13,14,15,16,17] and theoretical [18,19,25,26,27] studies on various DNA fragment models indicate that low-energy electrons may induce the DNA glycosidic bond cleavage through the mechanism of the dissociative electron attachment.

A study [28] examining the effects of UV radiation (140 nm) on condensed phase DNA cast films under vacuum conditions unveiled that the predominant consequence is the disruption of sugar C-O-C bonds, leading to C=O bond formation and phosphate group fragmentation. Additionally, this damage extends to thymine molecules engaged in Hoogsteen base pairing. The study of the UV photofragmentation of gas phase nucleotides indicated that the glycosidic bond cleavage is a possible mechanism for explaining the observation of base anion fragments [29]. The experimental study by Zheng et al. [13] showed that the low-energy electrons could cleave the thymidine glycosidic bond. They proposed that the excess low-energy electron probably locates on the antibonding orbitals related to the thymidine glycosidic bond, leading to the heterolytic cleavage of glycosidic bonds, finally generating the thymin-N1-yl anions and neutral 2-deoxyribose-C1(H)-yl radical. Gomes and co-workers [17] studied the energy thresholds of the DNA damage (including the phosphate group and base release) caused by UV within the range of 3.8–8.0 eV through X-ray photoelectron spectroscopy. The results showed that UV radiation can cause DNA damage via the formation of a transient negative ion at the initial step, releasing the DNA bases after breaking phosphate ester, requiring an energy above 4.2 eV (97.0 kcal/mol). Additionally, the generation of a transient anion may be an initial step during UV-induced phosphate breaking.

Gu and co-workers [18] chose 2′-deoxycytidine-3′-monophosphate as a model to study the inceptive mechanism of DNA strand damage caused by low-energy electrons using the B3LYP/DZP++ method. The results showed that, in the gas phase, the excess electron density of the radical anion generated by the low-energy electron attack is mainly situated on the pyrimidine rather than phosphate group. Furthermore, they [19,25,26] reported on the detailed mechanism of glycosidic bond cleavage using the models of pyrimidine nucleoside and nucleotide anion radicals. The free-energy barriers of breaking glycosidic bond are predicted to be about 18.0 and 21.2 kcal/mol for 2′-deoxyribothymidine and 2′-deoxyribocytidine, respectively, whereas they are about 21.1 and 26.2 kcal/mol for 2′-deoxythmidine-3′,5′-diphosphate and 2′-deoxycytidine-3′,5′-diphosphate radical anions, respectively. This indicates that, due to the lower activation energy, the thymine release is more prone to occur relative to the cytosine release. They [19] pointed out that the unpaired electron is positioned at the antibonding orbital of glycosidic bond exclusively at the transition state, differing from the experimental proposal [10] of a low-energy electron attaching directly to the antibonding orbital of the rupturing glycosidic bond. Li et al. [27] used the models of 2′-deoxyribothymidine and 2′-deoxyribocytidine to study the mechanism of DNA base release based on the B3LYP/6-31+G(d) method. The activation energy for 2′-deoxyribothymidine is 18.8 kcal/mol, which is a little higher for 2′-deoxyribocytidine (20.1 kcal/mol). This is qualitatively in line with the results reported by Gu et al. using the B3LYP/DZP++ method.

**Figure 1:**
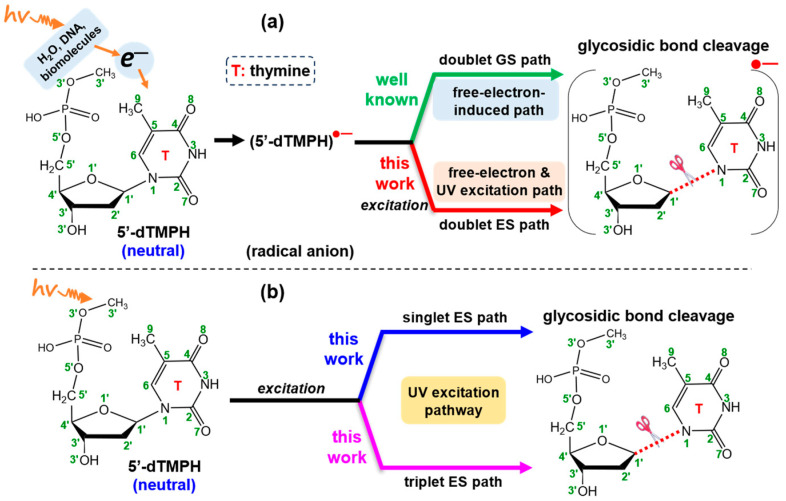
Molecular structure of 5′-dTMPH model, and possible pathways of breaking glycosidic bond, (**a**) free-electron-induced path and free-electron and UV excitation path, and (**b**) UV excitation pathway. The numbering of atoms is in green.

The previous studies [18,19,25,26,27] by other groups focused on the radical anion to analyze the free-electron-induced mechanism of glycosidic bond cleavage, which corresponds to the doublet GS path (green-colored line) in Figure 1a. To achieve a thorough understanding of the glycosidic bond cleavage mechanism, it is essential to consider the other possible pathways. For this purpose, we proposed and examined the following new reaction paths. First, in the radical anion, the glycosidic bond cleavage is also possible via excitation (red-colored line in Figure 1a). Second, the glycosidic bond of the neutral 5′-dTMPH may also be broken via excitation (blue- and magenta-colored lines in Figure 1b). If the direct UV excitation path is feasible, it should be simpler than the authorized electron-induced one.

Due to the limited theoretical study on UV-induced DNA glycosidic bond cleavage, our objective is to thoroughly explore this mechanism to achieve new insights into DNA glycosidic bond cleavage. Therefore, density functional theory (DFT) and time-dependent DFT (TDDFT) [30] were employed to study the glycosidic bond cleavage mechanism of neutral and radical anion 5′-dTMPH affected by UV radiation.

## 2. Results and Discussion

This section is divided into the following parts. The first, Section 2.1, shows the free-electron-induced path and free-electron and UV excitation path of glycosidic bond cleavage where the initial step is caused by the free electron addition to form the radical anion (Figure 1a). The second, Section 2.2, covers the UV excitation pathway of glycosidic bond cleavage, which is directly excited by UV light (Figure 1b). Finally, the overall mechanism of glycosidic bond cleavage is presented in Section 2.3.

The model in this study, 5′-dTMPH, consists of deoxyribose, thymine and a phosphate group attached at the C5′ atom of the sugar ring (Figure 1). In this model, a proton was added to the anionic phosphate group for system neutralization. The 5′-end phosphate group was terminated with a -CH_3_ group to provide a refined depiction of the implications arising from the 3′-5′ phosphodiester connection of DNA (Figure 1).

### 2.1. Free-Electron-Induced Path and Free-Electron and UV Excitation Path of C1′-N1 Bond Cleavage

**Figure 2:**
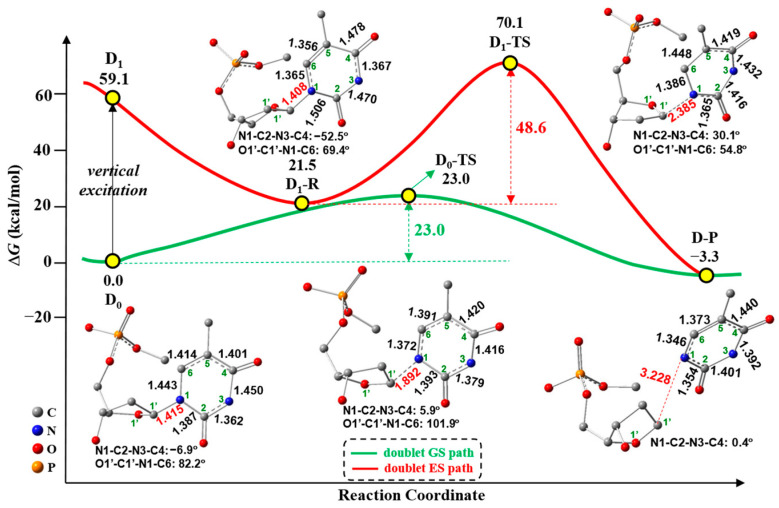
Gibbs free-energy profile of the C1′-N1 bond cleavage along free-electron-induced path (green-colored line) and free-electron and UV excitation path (red-colored line), including the GS and ES geometries (bond length in Å) where hydrogen atoms are not shown.

Figure 2 shows the possible paths of C1′-N1 bond cleavage with the schematic connecting curves between plots, including the corresponding GS and ES geometries for the free-electron-induced path (green-colored line) and free-electron and UV excitation path (red-colored line). The first step of these two paths is the formation of radical anion 5′-dTMPH, which results from the free electron addition to neutral 5′-dTMPH, as shown in Figure 1a. The first path (green-colored line) is the doublet GS path caused by the free electron, which corresponds to the widely studied mechanism [18,19,25,26,27] by other research groups. The second path (red-colored line) is the doublet ES path caused by the free electron together with the UV excitation of target radical anion 5′-dTMPH, which is proposed in this study.

**Figure 3:**
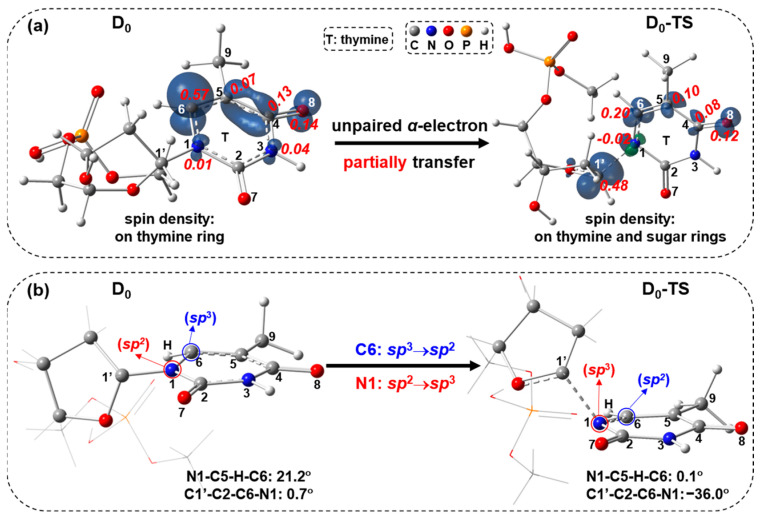
(**a**) Visualization of spin density ρ (ρ = ρ(α spin electron density) − ρ(β spin electron density); α in navy blue, β in green; isovalue = 0.01) with Mulliken spin-population values (decimals in red italics) for D_0_ and D_0_-TS, and (**b**) different view of D_0_ and D_0_-TS geometries.

For the doublet GS path, starting from D_0_ (D: doublet, D_0_: doublet GS), the C1′-N1 bond cleavage occurs via the transition state D_0_-TS (imaginary frequency: 592*i* cm^−1^, TS: transition state) to afford the doublet product D-P (P: product). In the D_0_-TS as shown in Figure 2, the C1′-N1 bond is elongated to 1.892 Å from 1.415 Å (D_0_). This step requires a free-energy barrier of 23.0 kcal/mol, and it is exergonic by 3.3 kcal/mol. This energy barrier is qualitatively consistent with the energy barriers (~20 kcal/mol) [9,27,31] using different DFT functionals and basis sets based on different research models when cleaving the C1′-N1 bond. Due to the low-energy barrier, the doublet GS path is feasible in kinetics. The spin-density maps of D_0_ and D_0_-TS with Mulliken spin-population values and a different view of D_0_ and D_0_-TS geometries are shown in Figure 3. The spin densities (Figure 3a) of D_0_ mainly focus on the C6 (0.57), C4 (0.13) and O8 (0.14), indicating that the unpaired single electron is mainly distributed on the thymine ring. In D_0_-TS, the spin densities mainly concentrate on C1′ (0.48), C6 (0.20) and O8 (0.12), revealing that the unpaired single electron is mainly distributed on the thymine and sugar rings. Compared with the spin-density distributions from D_0_ to D_0_-TS, it has a partial transfer of the unpaired α-electron from the thymine ring to sugar ring. Regarding the geometrical change towards TS, the C6 atom varies from the tetrahedral center to the trigonal planar center via *sp*^3^→*sp*^2^, and the N1 atom varies from the trigonal planar center to the tetrahedral center via *sp*^2^→*sp*^3^ (Figure 3b).

**Figure 4:**
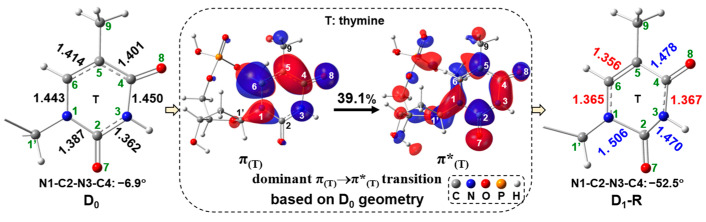
The π_(T)_→π*_(T)_ transition (molecular orbital (MO): isovalue = 0.03) based on the D_0_ geometry and the simplified D_0_ and D_1_-R geometries (bond length in Å). Percentage value represents the transition contribution.

Next, UV radiation can further excite the electron-induced radical anion D_0_. To confirm which transition is chosen at the first step, the spectrum and the main vertical parameters for radical anion 5′-dTMPH based on the D_0_ geometry are shown in Figure S1 and Table S1, respectively. The corresponding transitions with oscillator strength greater than 0.01 are shown in Figure S2. Among the low-lying excitations, the D_0_→D_1_ (D_1_: the 1st doublet ES) transition is chosen for the following discussions because it has a relatively large oscillator strength and a large impact on the C1′-N1 bond that is expected by sunlight UV irradiation (Figure S2). Initiating the doublet ES path is a vertical excitation from D_0_ to D_1_, with an associated free-energy requirement of 59.1 kcal/mol (Figure 2). Thereafter, the doublet ES reactant D_1_-R (R: reactant) is achieved by the geometrical optimization from the D_1_ structure for stabilizing the ES with a relative free energy of 21.5 kcal/mol, which is significantly lower than that of D_1_ (59.1 kcal/mol). To illuminate the geometric distinctions of D_1_-R relative to D_1_ geometry (same as D_0_ geometry), Figure 4 showcases the transition from π_(T)_ (π bonding orbital of thymine ring) to π*_(T)_ (π antibonding orbital of thymine ring) based on the D_0_ geometry and the corresponding simplified D_0_ and D_1_-R geometries. Compared to the D_0_ geometry, the N1-C2, C2-N3 and C4-C5 bond lengths in D_1_-R geometry are elongated 1.387→1.506 Å, 1.362→1.470 Å and 1.401→1.478 Å, respectively, while the N3-C4, C5-C6 and N1-C6 bond lengths in D_1_-R geometry are reduced 1.450→1.367Å, 1.414→1.356 Å and 1.443→1.365 Å, respectively. Only π_(T)_→π*_(T)_ transition with the contribution of 39.1% is displayed in Figure 4 as it is predominant, and Figure S2 displays the entire transitions. When an electron is excited from π_(T)_ to π*_(T)_, the enhancement of out-of-phase overlaps in π*_(T)_ promotes the elongation of N1-C2 and C2-N3, and the transformation from C4-C5 in-phase overlap (in π_(T)_) to out-of-phase overlap (in π*_(T)_) leads to the C4-C5 bond elongation. On the contrary, the transformation from out-of-phase overlap (in π_(T)_) to in-phase overlap (in π*_(T)_) results in the reduction of N1-C6 and N3-C4 bonds upon electronic excitation. Specially, the significant increase of N1-C2 bond from D_0_ to D_1_-R occurs, demonstrating this bond may be destroyed in D_1_-R.

**Figure 5:**
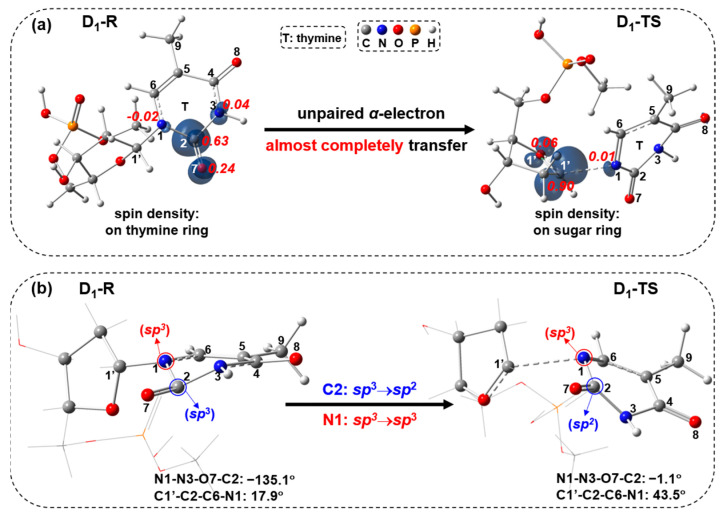
(**a**) Visualization of spin density ρ (ρ = ρ(α spin electron density) − ρ(β spin electron density); α in navy blue, β in green; isovalue = 0.01) with Mulliken spin-population values (decimals in red italics) for D_1_-R and D_1_-TS, and (**b**) different view of D_1_-R and D_1_-TS geometries.

The subsequent C1′-N1 bond cleavage takes place through the doublet ES transition state D_1_-TS (imaginary frequency: 352*i* cm^−1^), which is obtained by geometrical optimization, yielding the doublet product D-P (Figure 2). In the D_1_-TS, the C1′-N1 bond is stretched to 2.385 Å relative to 1.408 Å (D_1_-R), with a very high free-energy barrier of 48.6 kcal/mol, which is not favored in kinetics. As shown in Figure 2, a conical intersection (CI) between D_0_ and D_1_ potential energy surfaces near D_1__R might exist. The path along D_1_-R → D_0_/D_1_-CI → D_0_-TS → D-P could compete with the doublet ES path. In addition, the internal conversion (IC) from the higher doublet ESs to the D_1_ state can take place and subsequently return to D_0_ via the thymine ring puckered CIs [32,33,34,35,36], competing with the doublet ES path. Additionally, the D_1_-R geometry along the doublet ES path is similar to the hopping point with the stretched N1-C2 bond and the bent thymine ring, which is studied by Alexandrova et al. [37]. This hopping point can undergo the N1-C2 bond cleavage, which could compete with the C1′-N1 bond cleavage through the doublet ES path. The spin-density visualization of D_1_-R and D_1_-TS with Mulliken spin-population values and the different view of D_1_-R and D_1_-TS geometries are shown in Figure 5. The spin densities (Figure 5a) of D_1_-R are primarily centered at the C2 (0.63) and O7 (0.24), implying the unpaired single electron is delocalized on the thymine ring. For D_1_-TS, the spin density focuses on C1′ (0.90), revealing that the unpaired single electron is localized on the sugar ring. Compared with the spin-density distributions from D_1_-R to D_1_-TS, the unpaired α-electron almost completely transfers from the thymine ring to the sugar ring towards TS. Regarding geometries, the C2 atom changes from the tetrahedral to the trigonal planar via *sp*^3^→*sp*^2^, while the N1 atom keeps the tetrahedral center (Figure 5b).

### 2.2. UV Excitaiton Pathway of C1′-N1 Bond Cleavage

**Figure 6:**
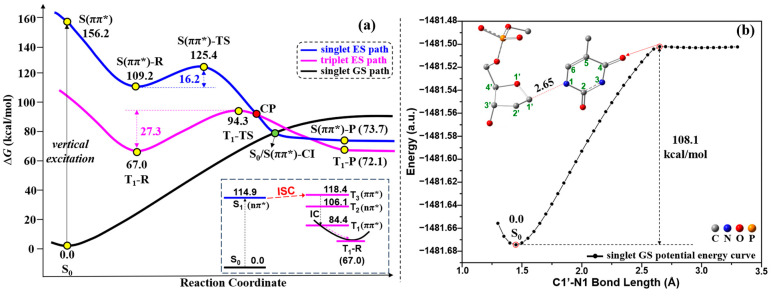
(**a**) Gibbs free-energy profile of the C1′-N1 bond cleavage for UV excitation pathway, and ISC from singlet to triplet states with excitation energies (kcal/mol), and (**b**) the singlet GS potential energy curve as the function of C1′-N1 bond length (Å).

**Figure 7:**
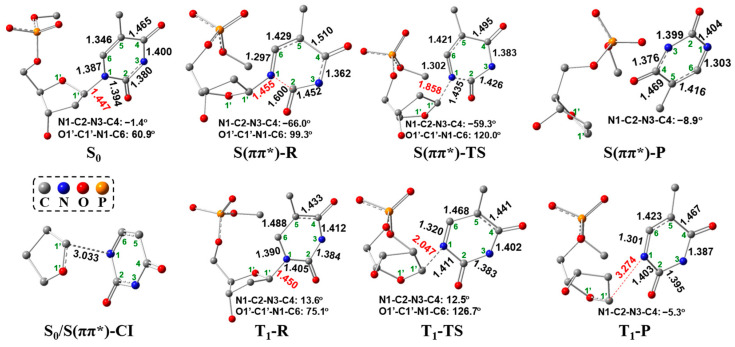
GS and ES geometries (bond lengths in Å) along UV excitation pathway where hydrogen atoms are not shown. S_0_/S(ππ*)-CI geometry is obtained based on CASSCF method using the simplified 5′-dTMPH model.

As discussed above, for the indirect free-electron-induced C1′-N1 bond cleavage, the capture of the free electron by neutral 5′-dTMPH to form the radical anion 5′-dTMPH is the first step. Unlike the electron-induced C1′-N1 bond cleavage, neutral 5′-dTMPH can be directly excited by UV radiation (Figure 1b). This process is simpler because UV light acts directly on the neutral 5′-dTMPH, without capturing the free electron to form the radical anion 5′-dTMPH. The possible UV excitation pathway is depicted in Figure 6a with the schematic connecting curves between plots. First, to confirm whether the C1′-N1 bond of neutral 5′-dTMPH is cleaved or not without the effect of UV light, many attempts were made to search for the transition state responsible for the C1′-N1 bond cleavage, but they failed. Then, the singlet GS potential energy curve is obtained via scanning the fixed C1′-N1 bond length at a range of values to predict the possible energy for C1′-N1 cleavage, as shown in Figure 6b. The result showed that the C1′-N1 bond cleavage requires a very high potential energy of 108.1 kcal/mol, indicating that this process without UV irradiation may be not favorable in kinetics. Under the UV radiation, on the other hand, a vertical excitation from the S_0_ (S: singlet, S_0_: singlet GS) to S(ππ*) (singlet ES with ππ* nature) of neutral 5′-dTMPH is perceived as the nascent step, as shown in Figure 6a. Here, the S(ππ*) along the singlet ES path is assigned based on the excitation nature ππ* because of the change of the energy orders of the ESs during the TDDFT optimization. The state with the excitation nature ππ* was followed during the calculations of TS and CI along the singlet ES path. To clearly clarify this point, the corresponding TS and CI, and others along the singlet ES path, were defined by tracking excitation nature ππ* in Figure 6a. To explain why the S_0_→S(ππ*) transition is chosen at this step, the spectrum and main vertical parameters for neutral 5′-dTMPH based on the S_0_ geometry are shown in Figure S3 and Table S2, respectively. The corresponding transitions with oscillator strength greater than 0.01 are shown in Figure S4. Furthermore, the comparison between the S_2_ geometry and S(ππ*)-R geometry is shown in Figure S5. Among the low-lying excitations, the S_0_→S(ππ*) transition is chosen at the first step because it has a relatively large oscillator strength and large effect on the C1′-N1 bond that is expected from the transition nature, and the corresponding S(ππ*)-R geometry resembles the hopping point with the elongated N1-C2 bond and bent thymine ring, which is observed in most of the trajectories by Alexandrova et al. when studying the nonadiabatic process of thymine 4H’-nucleoside [37]. Starting from S(ππ*), there are two possible paths to explain the C1′-N1 bond cleavage. One is the singlet ES path (blue-colored line in Figure 6a), and the other one is the triplet ES path (magenta-colored line in Figure 6a) from the singlet state via ISC (the inset at the bottom right corner of Figure 6a). The corresponding geometries along the singlet and triplet paths (yellow and green points) of Figure 6a are shown in Figure 7.

**Figure 8:**
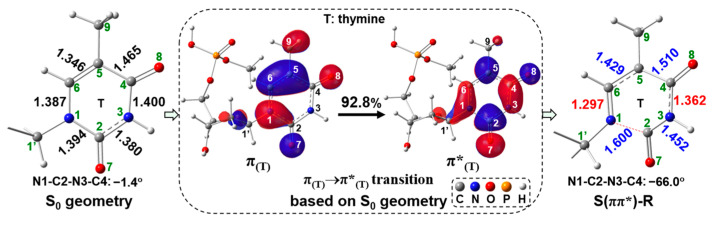
The π_(T)_→π*_(T)_ transition (MO: isovalue = 0.03) based on the S_0_ geometry, including the simplified S_0_ and S(ππ*)-R geometries (bond length in Å). Percentage value represents the transition contribution.

As for the singlet ES path based on our calculations, initially the intermediate S(ππ*)-R is obtained by geometrical optimizations with the geometry of the distorted thymine ring, in which the N1-C2-N3-C4 dihedral angle is changed from −1.4° to −66.0°, and N1-C2 and C2-N3 bond distances are elongated 1.394→1.600 Å and 1.380→1.452 Å (Figure 7), respectively. The S(ππ*)-R geometry is quantitatively in line with the hopping point with the stretched N1-C2 bond and the bent thymine ring that exists in most of the trajectories [37]. This hopping point is studied on the nonadiabatic process of thymine 4H’-nucleoside by Alexandrova et al. [37] From S_0_, an electron is excited to S(ππ*) state and then relaxed to the equilibrium geometry S(ππ*)-R keeping the ππ* transition nature. To expound the geometric variation of S(ππ*)-R relative to the S(ππ*) geometry (same as the S_0_ geometry), Figure 8 shows the molecular orbitals (MOs) based on the S_0_ geometry together with the simplified S_0_ and S(ππ*)-R geometries. Based on the S_0_ geometry, the transition from π_(T)_ to π*_(T)_ in S_0_→S(ππ*) excitation has an electronic transition contribution of 92.8% (see also Table S2). Upon the π_(T)_→π*_(T)_ excitation, the N1-C2, C4-C5 and C5-C6 bonds are elongated due to the in-phase overlap at π_(T)_ and strong out-of-phase at π*_(T)_ on those bonds. The C2-N3 bond is also elongated because of the enhanced out-of-phase at π*_(T)_ when the electron is excited to π*_(T)_. On the other hand, the N1-C6 bond is reduced due to the out-of-phase overlap at π_(T)_ and the in-phase overlap at π*_(T)_ on this bond. The N3-C4 bond is also reduced because of the reinforced in-phase overlap at π*_(T)_ upon the electronic excitation to π*_(T)_. In particular, the N1-C2 bond is largely stretched from 1.394 Å (S_0_) to 1.600 Å (S(ππ*)-R), indicating that this N1-C2 bond may be broken in the S(ππ*)-R geometry. Additionally, owing to be free from the geometric constraints of the quinoid-like S_0_ structure, the thymine plane is largely twisted in S(ππ*)-R, in which the C2 atomic configuration has a more significant change than the other atoms of thymine, from the trigonal planar to tetrahedral, signifying the formation of the non-plane thymine ring in S(ππ*)-R. Next, we tried to search for the singlet ES transition state (corresponding to S(ππ*)-TS in Figure 7) for cleaving the C1′-N1 bond from the S(ππ*)-R geometry.

**Figure 9:**
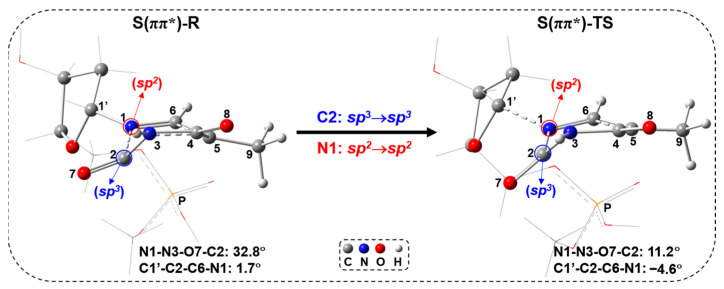
Geometries of S(ππ*)-R and S(ππ*)-TS.

The singlet ES transition state S(ππ*)-TS is obtained by geometrical optimization, which is featured by the presence of one single imaginary frequency (849*i* cm^−1^) referring to the stretching/shrinking along the C1′-N1 and N1-C2 bond directions. In S(ππ*)-TS, as shown in Figure 7, the C1′-N1 bond distance increases to 1.858 Å from 1.455 Å (S(ππ*)-R), while the N1-C2 bond distance decreases to 1.435 Å from 1.600 Å (S(ππ*)-R). The largely elongated C1′-N1 bond and considerably reduced N1-C2 bond in S(ππ*)-TS suggest that the C1′-N1 bond scission occurs after the damage of the thymine ring. This step requires a low-energy barrier of 16.2 kcal/mol (Figure 6a). Furthermore, according to the study by Alexandrova et al. [37], the above-mentioned hopping point can go to the structures with the N1-C2 bond cleavage, which could be a competitive path with the C1′-N1 bond cleavage via the singlet ES path. Additionally, the IC can also occur from the higher ESs to the S_1_ state, leading to a return to S_0_ through the ring-puckered Cis, which are widely studied [32,33,34,35,36]. Also, this IC process can be competitive with the singlet ES path. To obtain a comprehensive understanding, these competitive paths will be examined in our next study. Figure 9 shows the comparison between S(ππ*)-R and S(ππ*)-TS. From S(ππ*)-R to S(ππ*)-TS, there is no electron transfer from thymine to sugar in the population analysis, and the C2 and N1 atoms keep the tetrahedral (*sp*^3^) and triangle planar (*sp*^2^) in S(ππ*)-TS, respectively, with respect to the geometrical change (Figure 9).

**Figure 10:**
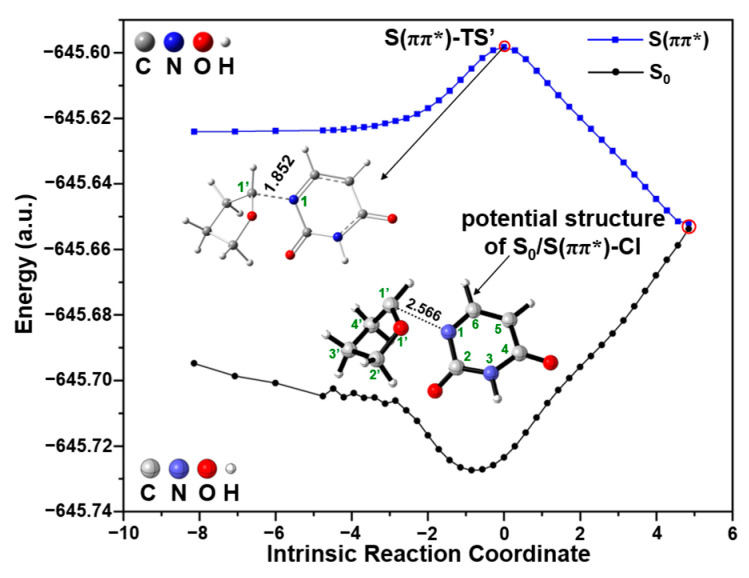
The potential S_0_/S(ππ*)-CI structure between S_0_ and S(ππ*) from the IRC calculation of S(ππ*)-TS’ using simplified 5′-dTMPH model based on (TD-)M06-2X/6-31G(d) method.

After that, it should be noted that, from S(ππ*)-TS to S(ππ*)-P, there may exist a S_0_/S(ππ*)-CI between the singlet GS and singlet ES potential energy surfaces, as shown in Figure 6a. To obtain the potential S_0_/S(ππ*)-CI structure between S_0_ and S(ππ*), Figure 10 shows the IRC calculation of S(ππ*)-TS’ at the TD-M06-2X/6-31G(d) level using the simplified 5′-dTMPH model. For each point along the singlet ES energy path, the single-point potential energy is performed in the singlet GS state, from which the potential structure of S_0_/S(ππ*)-CI is estimated. The excitation energy of the potential S_0_/S(ππ*)-CI structure is almost zero eV. Then, the more accurate S_0_/S(ππ*)-CI geometry (Figure 7) was obtained based on the CASSCF method starting from the above potential structure. From this S_0_/S(ππ*)-CI, it is possible to reach the product of C1′-N1 bond cleavage or return the S_0_. One possibility is the formation of the singlet product S(ππ*)-P along the singlet ES potential energy surface, which is highly exergonic by 35.5 kcal/mol relative to S(ππ*)-R (Figure 6a). The second possibility is to return the S_0_ along the singlet GS potential energy surface. In addition, there might be an alternative possibility to reach the triplet product T_1_-P (T: triplet; T_1_: the 1st triplet ES, 72.1 kcal/mol) through the crossing point (CP) between the singlet ES and triplet ES surfaces, which have reduced lower free energy compared to S(ππ*)-P (73.7 kcal/mol).

Alternatively, the C1′-N1 bond cleavage of the 5′-dTMPH model can be achieved through the triplet ES path starting from the triplet reactant T_1_-R. Concerning the triplet ES path shown in Figure 6a, ISC as the first step may occur from the singlet ES to triplet ES potential energy surfaces as shown in the inset at the bottom right corner of Figure 6a (see also Figure S6). It can be seen that the T_3_ excitation energy is close to that of the S_1_ state. Owing to the energy near-degeneracy between the S_1_ and T_3_ states, it is possible to undergo the ISC from the S_1_ to T_3_ states. Consequently, the T_3_ state goes to the T_1_ state via IC, further relaxing to the T_1_-R (Figure 6a). In accordance with the EI-Sayed rule [38], the transition from the S_1_ (^1^nπ*) state to an excited triplet state necessitates that the excited triplet state shows significant ππ* character, enabling the correlated spin–orbit coupling (SOC). Featuring ππ* nature, the T_3_ state displays an energy gap of 3.5 kcal/mol relative to that of the S_1_ state. The near-degeneracy of energy between singlet and triplet states and a large SOC constant of 21.9 cm^−1^ (Figure S6) lead to efficient ISC. Therefore, ISC occurs from the S_1_ (^1^nπ*) state to the T_3_ (^3^ππ*) state. Besides the energy gap and SOC constant, the IC and ISC rates should be studied, since the ISC is impossible when the IC rates are much higher than the ISC rates. The IC and ISC rates will be analyzed in our next study to better understand the studied mechanism. Hereafter, the T_3_ state goes to the T_1_ state via IC, relaxing to the equilibrium geometry T_1_-R along the triplet path. The thymine ring of T_1_-R is a little bent along the N3-C6 line (N1-C2-N3-C4 dihedral angle: 13.6°) relative to the S(ππ*) geometry (same as the S_0_ geometry), as shown in Figure 7. The relative free energy of T_1_-R is 67.0 kcal/mol, which is highly exergonic compared to that of the S(ππ*) state (156.2 kcal/mol), as displayed in Figure 6a. Additionally, this free energy of T_1_-R (67.0 kcal/mol) is much lower than that of S(ππ*)-R (109.2 kcal/mol). The results showed that it may be more prone to reach the stable triplet ES reactant T_1_-R from the singlet ES potential energy surface via ISC.

**Figure 11:**
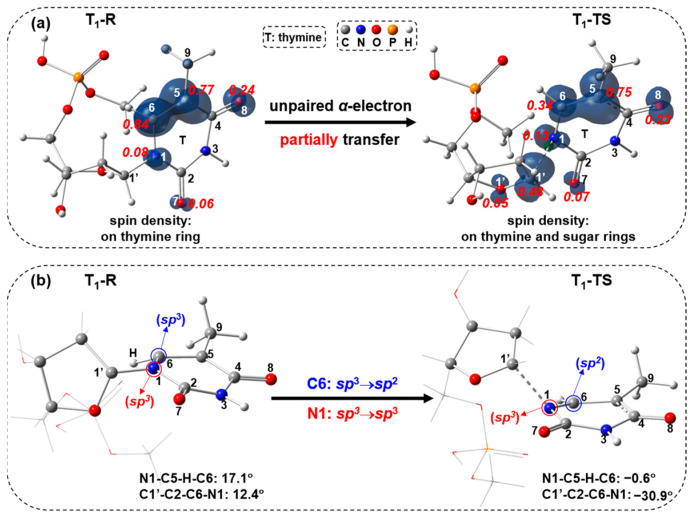
(**a**) Visualization of spin density ρ (ρ = ρ(α spin electron density) − ρ(β spin electron density); α in navy blue, β in green; isovalue = 0.01) with Mulliken spin-population values (decimals in red italics) for T_1_-R and T_1_-TS, and (**b**) different view of geometries of T_1_-R and T_1_-TS.

Subsequently, the C1′-N1 bond cleavage occurs through the triplet ES transition state T_1_-TS with a single imaginary frequency of 524*i* cm^−1^. As shown in T_1_-TS in Figure 7, the C1′-N1 bond distance has a large increase to 2.047 Å, keeping the N1-C2-N3-C4 dihedral angle similar to T_1_-R. The free-energy barrier of T_1_-TS is calculated to be 27.3 kcal/mol compared to T_1_-R. This triplet ES path is feasible considering the ISC occurrence. The spin-density visualization of T_1_-R and T_1_-TS with Mulliken spin-population values and different view of T_1_-R and T_1_-TS geometries are shown in Figure 11. The spin densities (Figure 11a) of T_1_-R are distributed at the C6 (0.84), C5 (0.77) and O8 (0.24), implying the unpaired single electron is delocalized on thymine rings. For T_1_-TS, the spin density focuses on the C1′ (0.48), C6 (0.34), C5 (0.75) and O8 (0.23), revealing that the unpaired single electron is delocalized on thymine and sugar rings. Compared to the spin-density distribution of T_1_-R, the unpaired α-electron of T_1_-TS partially transfers from thymine ring to sugar ring. As for the geometrical change, the C6 atom varies from tetrahedral to triangle planar via *sp*^3^→*sp*^2^, and the N1 atom keeps the tetrahedral center (Figure 11b).

Finally, as well as the singlet path, the triplet product T_1_-P may also be generated with a long C1′-N1 bond distance of 3.274 Å or go back to the S_0_ along the GS potential energy surface via the CP and S_0_/S(ππ*)-CI between the singlet GS and triplet ES energy surfaces, as shown in Figure 6a.

### 2.3. Overall Mechanism of C1′-N1 Bond Cleavage

**Figure 12:**
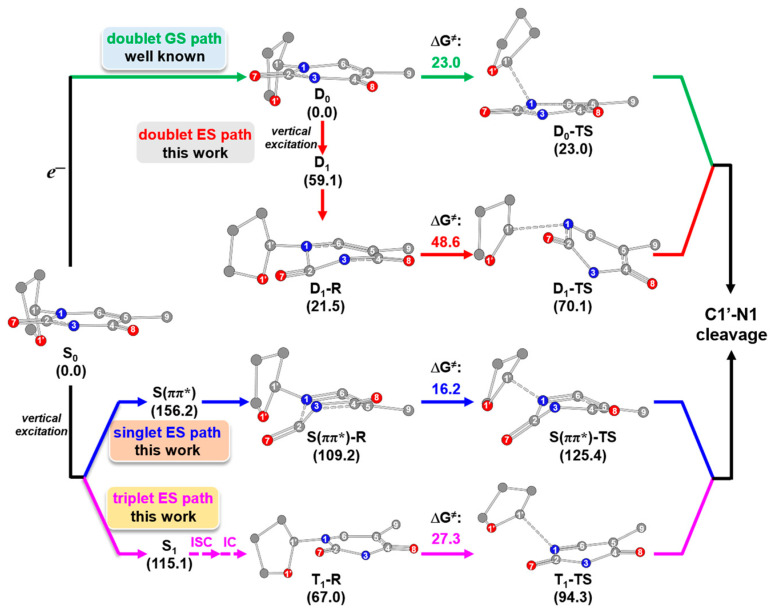
Overall mechanism of C1′-N1 bond cleavage for 5′-dTMPH model, including the simplified geometries and their Gibbs free energies (kcal/mol). ΔG^≠^ represents the difference in free energy from the reactant to the transition state.

Collectively, Figure 12 displays the overall paths for C1′-N1 bond cleavage. Owing to a low free-energy barrier of 23.0 kcal/mol, the authorized doublet GS path caused by the free electron is a feasible path to cleave the C1′-N1 bond. However, the doublet ES path caused by the free electron together with UV excitation, which is proposed in this work, is unfavorable in kinetics due to a very high-energy barrier of 48.6 kcal/mol. Compared to the above two paths, the direct UV-induced singlet ES path proposed in this work is more feasible, given the lower free-energy barrier of 16.2 kcal/mol. Moreover, the triplet path proposed in this study, having a free-energy barrier of 27.0 kcal/mol, is also possible because of potential ISC from singlet to triplet states.

From the results, it can be concluded that the DNA glycosidic bond cleavage by our proposed direct UV excitation paths, especially the UV-induced singlet ES path, can be a more feasible and pivotal reaction in DNA damage in addition to the authorized indirect free-electron-induced path.

## 3. Computational Details

DFT and TDDFT calculations were performed on the 5′-dTMPH model (Figure 1) to explore the mechanisms of photoinduced DNA glycosidic bond cleavage. The initial structure of the 5′-dTMPH model is extracted from the crystal structure of a B-DNA dodecamer [39]. The 5′-dTMPH model used here has some limitations, such as the lack of the other bases, the chemical interactions between different bases and the double-helix structure, which could affect the mechanism. However, considering whether the geometry optimizations of ESs can be performed using the current TDDFT method, a small model should be used at the initial stage. Fully geometry optimizations for the GSs and ESs, including the reactants, transition states and products, were carried out without any constraints at M062X [40]/6-31G(d) and TD-M06-2X/6-31G(d) levels, respectively. Geometry optimizations for the singlet ES path were performed at the RM06-2X/6-31G(d) level, and for the triplet ES and doublet paths, they were made at the UM06-2X/6-31G(d) level. Previously, Kumar et al. [41] found that using TD-BHandHLYP with 50% HF exchange instead of TD-B3LYP [42,43,44] with 20% HF exchange can better describe the excitation of radical anion 5′-dTMPH when studying the electron-induced C5′-O5′ bond cleavage. Additionally, Chen and co-workers [31] conducted a benchmark study on the energy barrier of C3′-O3′, C5′-O5′ and C1′-N1 bond cleavages for the models of thymine nucleotides and nucleoside radical anions using a series of DFT functionals. The results indicate that CAM-B3LYP [45], ωB97XD [46] and M06-2X functionals are appropriate for modeling such type of bond breaking mechanism, and they have the errors within 1.5 kcal/mol compared to the CCSD(T) results. Therefore, M06-2X with 54% HF exchange, which is similar as BH and HLYP with 50% HF exchange was used in this work. Based on the above GS and ES geometries after optimization, M062X/6-311+G(d) and TD-M06-2X/6-311+G(d) methods were employed to obtain the single point energies for GSs and ESs, respectively. Frequency analyses were made using the M06-2X/6-31G(d) method to characterize the minima (no imaginary frequency) and transition states (one imaginary frequency) on the potential energy surfaces. Gibbs free energies including the thermal corrections were used, which were calculated under standard conditions of 298.15 K and 1 atm. Intrinsic reaction coordinate (IRC) [47] calculations were made to all the transition states to verify the connection between the reactant and product. The gas-phase free-energy calculations were utilized in this study because the experiment [13] focused on the glycosidic bond cleavage for the unsolvated thymidine molecules, which may be used to exclude the influence from the surrounding solvated molecules. GaussView 6.1.1 [48] and CYLview [49] visualization programs were employed to illustrate the 3D optimized structures. Electronic structure calculations were accomplished using Gaussian 16 [50] program package.

The S_0_/S(ππ*)-CI structure in Figure 6a and Figure 7 is obtained by combining with ORCA 5.0.4 [51] software and Gaussian 16 program. To evaluate a more accurate S_0_/S(ππ*)-CI structure using the complete active space self-consistent field (CASSCF) [52] method, the simplified 5′-dTMPH model was adopted, in which the -CH_2_PO_4_CH_3_ group at C4′ atom, the -OH group at C3′ atom and the -CH_3_ group at C5 atom were replaced by hydrogen atoms. Firstly, the potential S_0_/S(ππ*)-CI structure was evaluated from the IRC calculation of S(ππ*)-TS’. Secondly, starting from the above potential structure, a rough S_0_/S(ππ*)-CI structure was estimated at the B3LYP-D3BJ [53,54]/6-31G(d) level based on the spin–flip (SF)-TDDFT method using loose optimization criteria. Thirdly, based on the rough S_0_/S(ππ*)-CI structure, a more accurate S_0_/S(ππ*)-CI structure is obtained based on the CASSCF(6,6)/6-31G(d) method.

## 4. Conclusions

In the current work, DNA glycosidic bond breakage using the 5′-dTMPH model under the influence of UV light was studied based on the quantum chemical method. The potential energy curve for the singlet GS state as a reference indicates that, without UV light, the predicted energy is too high to cleave the glycosidic bond. In this study, the following four different paths for glycosidic bond cleavage were examined to understand the UV light effects. That is, under UV radiation, the glycosidic bond cleavage may take place through the authorized indirect free-electron-induced path (doublet GS path), free-electron and UV excitation path (doublet ES path) or direct UV excitation pathway. The doublet GS path is widely studied by other groups, while the doublet ES path is proposed in this study. The UV excitation pathway, including the singlet ES and triplet ES paths, is also proposed in this work.

The calculational results indicate that the π_(T)_→π*_(T)_ transition has a large effect on the C1′-N1 bond cleavage because π_(T)_ and π*_(T)_ have MO coefficients on the N1 atom or its nearby C1′ atoms, or both. For the process of C1′-N1 bond cleavage, the doublet ES path has a very high-energy barrier of 48.6 kcal/mol that is kinetically unfavorable. The authorized doublet GS path and the triplet ES path have the lower-energy barriers of 23.0 and 27.3 kcal/mol that are feasible in kinetics, respectively. Relative to the energy barriers of the other three paths, the singlet ES path in our proposed direct UV path has the lowest energy barrier of 16.2 kcal/mol, indicating that this path is more feasible than the others.

This research offers a comprehensive analysis of DNA glycosidic bond cleavage, highlighting the major role of direct UV excitation in this process. It is expected to yield key insights for developing the glycosidase-activated prodrugs that exploit glycosidic bond cleavage to release active anticancer agents specially at tumor sites [55].

## Data Availability

The data presented in this work are available in the article and Supplementary Materials.

## Supplementary Materials

The following supporting information can be downloaded at: https://www.mdpi.com/article/10.3390/molecules29163789/s1. Figure S1: Spectrum of radical anion 5′-dTMPH based on the D_0_ geometry; Table S1: Main parameters for the vertical excitations (UV–Vis absorption) of radical anion 5′-dTMPH based on the D_0_ geometry; Figure S2: D_0_→D_1_, D_0_→D_2_ and D_0_→D_5_ transitions (MO: isovalue = 0.03) based on the D_0_ geometry; Figure S3: Spectrum of neutral 5′-dTMPH based on the S_0_ geometry; Table S2: Main parameters for the vertical excitations (UV–Vis absorption) of neutral 5′-dTMPH based on the S_0_ geometry; Figure S4: S_0_→S_2_, S_0_→S(ππ*) and S_0_→S_5_ transitions (MO: isovalue = 0.03) based on the S_0_ geometry; Figure S5: Comparison between S_2_ geometry and S(ππ*)-R geometry. Figure S6: ISC from singlet to triplet states with the excitation energies (kcal/mol), the calculated SOC constant (cm^−1^) at B3LYP-D3BJ/6-31G(d) level using ORCA 5.0.4, and the S_1_-T_3_ energy gap (kcal/mol) for ISC.
