# Supplementary material for: A Theoretical Exploration of the Photoinduced Breaking Mechanism of the Glycosidic Bond in Thymine Nucleotide
Source: Molecules. 2024 Aug 10;29(16):3789. doi: 10.3390/molecules29163789 (PMC11357666; doi:10.3390/molecules29163789)
Supplement: Supplementary file 1 [file molecules-29-03789-s001.zip › molecules-3104171-supplementary.pdf]

**Supplementary Information**

# A Theoretical Exploration of the Photoinduced Breaking Mechanism of the Glycosidic Bond in Thymine Nucleotide

Xiao Huang [1], Yuuichi Orimoto [2] and Yuriko Aoki [1,2,*]

[1] Department of Interdisciplinary Engineering Sciences, Chemistry and Materials Science, Interdisciplinary Graduate School of Engineering Sciences, Kyushu University, 6-1 Kasuga Park, Fukuoka 816-8580, Japan; huang.xiao.932@s.kyushu-u.ac.jp

[2] Department of Material Sciences, Faculty of Engineering Sciences, Kyushu University, 6-1 Kasuga Park, Fukuoka 816-8580, Japan; orimoto.yuuichi.888@m.kyushu-u.ac.jp

* Correspondence: aoki.yuriko.397@m.kyushu-u.ac.jp

# Table of Contents

**1. Additional Figures**······S3

**1.1 Spectrum and main parameters of vertical excitation of radical anion 5′-dTMPH**······S3

**1.2 Spectrum and main parameters of vertical excitation of neutral 5′-dTMPH**······S6

**2. References**······S11

**3. Coordinates**······S12

# 1. Additional Figures

## 1.1 Spectrum and main parameters of vertical excitation of radical anion 5′-dTMPH

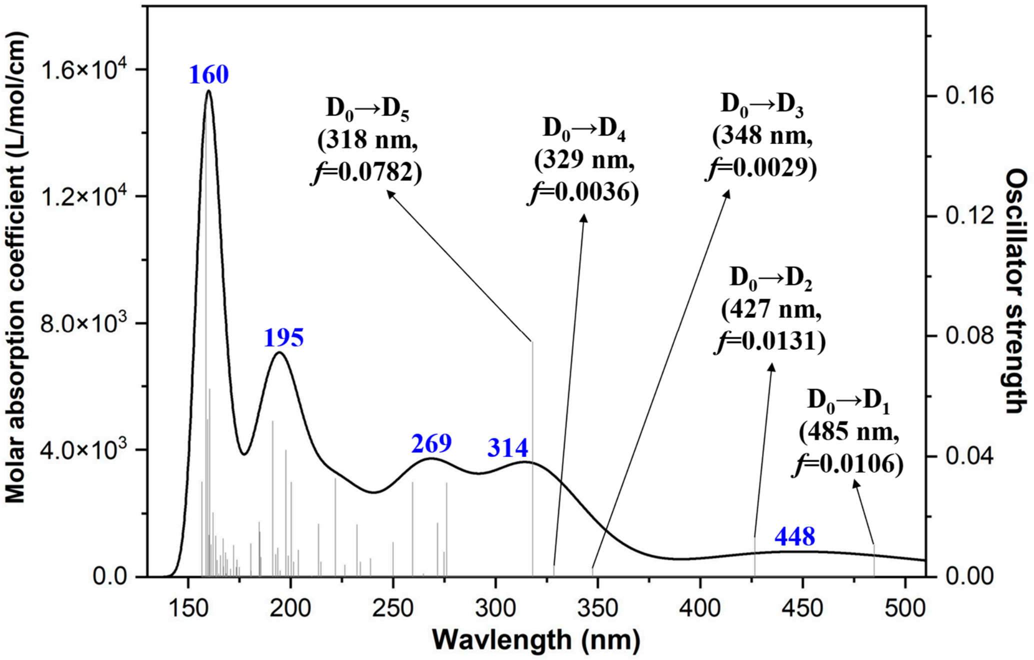


**Figure S1. Spectrum of radical anion 5′-dTMPH based on the $D_0$ geometry. (*f*: Oscillator strength)**

**Table S1. Main parameters for the vertical excitations (UV-Vis absorption) of radical anion 5′-dTMPH based on the $D_0$ geometry.**

| electronic transition | energy (eV) | λ (nm) | *f* | contributions | transition | assignment |
|---|---|---|---|---|---|---|
| $D_0 \rightarrow D_1$ | 2.56 | 485 | 0.0106 | 39.1% | S→L+3 | $\pi_{(T)} \rightarrow \pi^*_{(T)}$ |
| | | | | 25.1% | S→L | $\pi_{(T)} \rightarrow \sigma^*_{(PO4-)}$ |
| | | | | 15.7% | S→L+1 | $\pi_{(T)} \rightarrow \sigma^*_{(sugar)}$ |
| | | | | 11.3% | S→L+2 | $\pi_{(T)} \rightarrow \sigma^*_{(C\text{-}H\ of\ phosphate)}$ |
| $D_0 \rightarrow D_2$ | 2.91 | 427 | 0.0131 | 54.1% | S→L | $\pi_{(T)} \rightarrow \sigma^*_{(PO4-)}$ |
| | | | | 37.5% | S→L+3 | $\pi_{(T)} \rightarrow \pi^*_{(T)}$ |
| $D_0 \rightarrow D_3$ | 3.57 | 348 | 0.0029 | 80.1% | S→L+1 | $\pi_{(T)} \rightarrow \sigma^*_{(sugar)}$ |
| | | | | 11.5% | S→L+3 | $\pi_{(T)} \rightarrow \pi^*_{(T)}$ |
| $D_0 \rightarrow D_4$ | 3.77 | 329 | 0.0036 | 65.2% | S→L+2 | $\pi_{(T)} \rightarrow \sigma^*_{(phosphate)}$ |
| | | | | 16.2% | S→L | $\pi_{(T)} \rightarrow \sigma^*_{(PO4-)}$ |
| $D_0 \rightarrow D_5$ | 3.90 | 318 | 0.0782 | 52.6% | H→S | $\pi_{(T)} \rightarrow \pi^*_{(T)}$ |
| | | | | 28.7% | H→L | $\pi_{(T)} \rightarrow \pi^*_{(T)} \& \sigma^*_{(PO4-)}$ |

*f*: Oscillator strength S: Singly Occupied Molecular Orbital (SOMO) H: Highest Occupied Molecular Orbital (HOMO)

L: Lowest Unoccupied Molecular Orbital (LUMO) contributions: only the transition contribution greater than 10% are shown here.

T: thymine

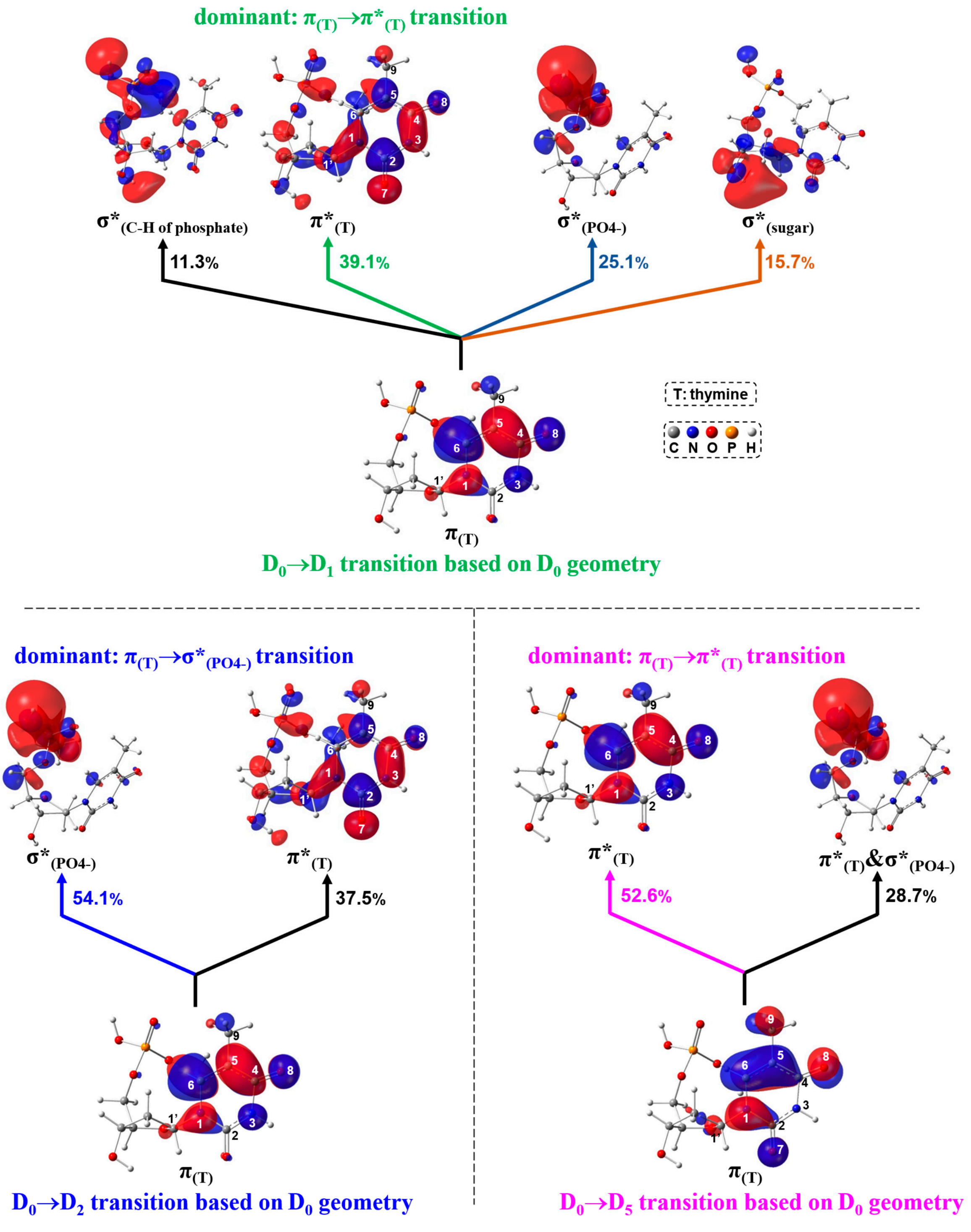


**Figure S2.** $D_0 \rightarrow D_1$, $D_0 \rightarrow D_2$ and $D_0 \rightarrow D_5$ transitions (MO: isovalue = 0.03) based on the $D_0$ geometry. (Only the transitions with the oscillator strength greater than 0.01 and the transition contribution greater than 10% are shown)

To explain why $D_0 \rightarrow D_1$ transition is chosen at the first step, the spectrum of radical anion 5′-dTMPH is shown in Figure S1, and the main parameters for the vertical excitations are shown in Table S1. Corresponding to the assignment of Table S1, Figure S2 shows the transitions based on the $D_0$ geometry in which only the transitions with oscillator strength greater than 0.01 and the transition contribution greater than 10% are displayed. The first five low-lying excitations are examined, given that absorption spectra are usually dominated by the first few excited states.

Firstly, $D_0 \rightarrow D_3$ and $D_0 \rightarrow D_4$ transitions are excluded due to the very small oscillator strengths. Secondly, $D_0 \rightarrow D_2$ transition has a dominant $\pi_{(T)} \rightarrow \sigma^*_{(PO4-)}$ transition, where $\pi_{(T)}$ has MO coefficients on thymine N1 atom and sugar C1' atom, but the MO coefficients of $\sigma^*_{(PO4-)}$ concentrate on $PO_4^-$ group. It indicates that only one factor, that is the decrease in bonding nature by excitation, is included to impact the C1'-N1 bond, leading to a little effect on the focused C1'-N1, as shown in Figure S2. Thirdly, for $D_0 \rightarrow D_1$ and $D_0 \rightarrow D_5$ transitions, both of them have relatively large oscillator strengths, and the dominant transition features $\pi_{(T)} \rightarrow \pi^*_{(T)}$ where $\pi_{(T)}$ and $\pi^*_{(T)}$ have MO coefficients on N1 or C1' atoms or both. It means that two factors, that is the decrease in bonding nature and the increase in antibonding nature by excitation, are contained to affect the C1'-N1 bond, indicating that these two transitions have the large influence on the focused C1'-N1 bond. As discussed above, the transition that involves one factor affecting the C1'-N1 bond is weaker compared to those involving two factors. Both cases should be analyzed because they both can affect the C1'-N1 bond. However, at the first step, this study only focuses on the more important transitions involving two factors, that is $D_0 \rightarrow D_1$ and $D_0 \rightarrow D_5$ transitions.

Here, $D_0 \rightarrow D_1$ and $D_0 \rightarrow D_5$ transitions have different absorption wavelengths. As we all known, the range of UV light from sunlight that reaches the earth mainly includes UVA (320-400 nm) and UVB (280-320 nm). UVA and UVB account for about 95% and 5% of the UV radiation reaching the earth, respectively. The absorption wavelength for $D_0 \rightarrow D_1$ transition is 485 nm which is close to UVA, while that for $D_0 \rightarrow D_5$ transition is 318 nm which is close to UVB. That means for UV radiation on the earth, $D_0 \rightarrow D_1$ transition is more important than $D_0 \rightarrow D_5$ transition. So, in this study, $D_0 \rightarrow D_1$ transition is chosen to do further discussions.

To confirm whether the $\pi_{(T)} \rightarrow \pi^*_{(T)}$ transition based on the $D_0$ geometry is the dominant transition or not as shown in Figure 4 of the main text, the $D_0 \rightarrow D_1$ transition based on $D_0$ geometry including the $\pi_{(T)} \rightarrow \pi^*_{(T)}$ transition is displayed in Figure S2. It can be seen that the $\pi_{(T)} \rightarrow \pi^*_{(T)}$ transition based on the $D_0$ geometry is the predominant transition, because it has a higher transition contribution 39.1% than other transitions.

## 1.2 Spectrum and main parameters of vertical excitation of neutral 5′-dTMPH

To better discuss the singlet ES path (see Figure 6a) below and in the main text, at first, the names of TS, CI, and others along the singlet ES path should be explained. Owing to the alteration of the ES energy orders during the TDDFT optimization, the S($\pi\pi^*$) along the singlet ES path is specified in terms of the excitation nature $\pi\pi^*$. Then, in the TS and CI calculations along the singlet ES path, the state with the excitation nature $\pi\pi^*$ was tracked. However, at the vertical excitation step, among the five low-lying excitations as shown in Table S2, both the 2$^{nd}$ singlet ES (energy: 5.44 eV) and the 4$^{th}$ singlet ES (energy: 6.76 eV) have the excitation nature $\pi\pi^*$. To clearly distinguish from these two excitations, only the 4$^{th}$ singlet ES is represented using the S($\pi\pi^*$) state based on its excitation nature $\pi\pi^*$ to clarify the change of the ESs energy orders during the TDDFT optimizations, while the 2$^{nd}$ singlet ES is represented using the $S_0 \rightarrow S_2$ transition, as shown in Figures S3-S4 and Table S2. It is because the 4$^{th}$ singlet ES is more important than the 2$^{nd}$ singlet ES at the first step, and the detailed reasons are shown in the following parts. Following the state with excitation nature $\pi\pi^*$, the corresponding reactant, TS, CI, and product along the singlet ES path are represented using the S($\pi\pi^*$)-R, S($\pi\pi^*$)-TS, $S_0$/S($\pi\pi^*$)-CI, and S($\pi\pi^*$)-P, respectively, as show in Figure 6a of the main text.

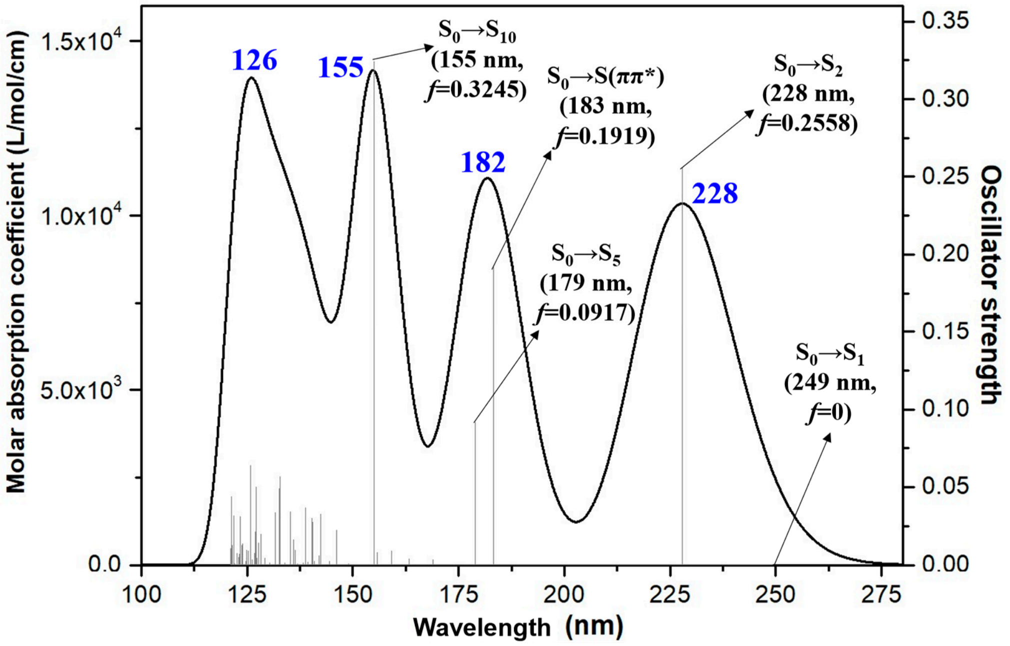


**Figure S3. Spectrum of neutral 5′-dTMPH based on the $S_0$ geometry. (*f*: Oscillator strength)**

**Table S2. Main parameters for the vertical excitations (UV-Vis absorption) of neutral 5′-dTMPH based on the $S_0$ geometry.**

| electronic transition | energy (eV) | λ (nm) | $f$ | contributions | transition | assignment |
|---|---|---|---|---|---|---|
| $S_0 \to S_1$ | 4.98 | 249 | 0 | 68.3%<br>10.4% | H-1→L<br>H-3→L | $n_{(O\ of\ T)}\&\sigma_{(N4\text{-}C5)} \to \pi^*_{(T)}$<br>$n_{(O\ of\ T)}\&\sigma(\text{sugar}) \to \pi^*_{(T)}$ |
| $S_0 \to S_2$ | 5.44 | 228 | 0.2558 | 96.0% | H→L | $\pi_{(T)} \to \pi^*_{(T)}$ |
| $S_0 \to S_3$ | 6.33 | 196 | 0.0001 | 35.1%<br>24.4%<br>17.1% | H-1→L+1<br>H-5→L+1<br>H-4→L+1 | $n_{(O\ of\ T)}\&\sigma_{(N4\text{-}C5)} \to \pi^*_{(T)}$<br>$n_{(O\ of\ T)}\&\sigma_{(sugar)} \to \pi^*_{(T)}$<br>$n_{(O\ of\ T)} \to \pi^*_{(T)}$ |
| $S_0 \to S(\pi\pi^*)$ | 6.76 | 183 | 0.1919 | 92.8% | H→L+1 | $\pi_{(T)} \to \pi^*_{(T)}$ |
| $S_0 \to S_5$ | 6.93 | 179 | 0.0917 | 93.5% | H-2→L | $p_{(N3,O7\&O8\ of\ T)} \to \pi^*_{(T)}$ |

$f$: Oscillator strength  H: HOMO  L: LUMO  contributions: only the transition contribution greater than 10% are shown here.

T: thymine

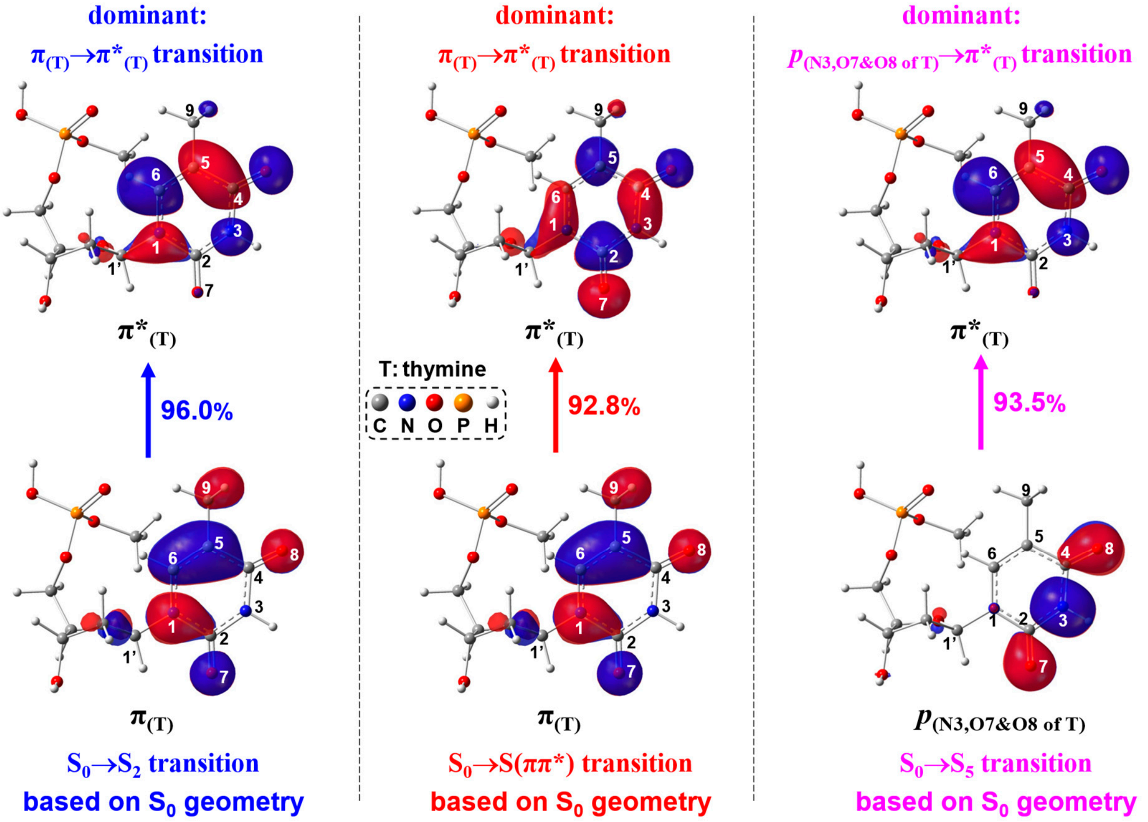


**Figure S4.** $S_0 \to S_2$, $S_0 \to S(\pi\pi^*)$ and $S_0 \to S_5$ **transitions (MO: isovalue = 0.03) based on the** $S_0$ **geometry. (Only the transitions with the oscillator strength greater than 0.01 and the transition contribution greater than 10% are shown)**

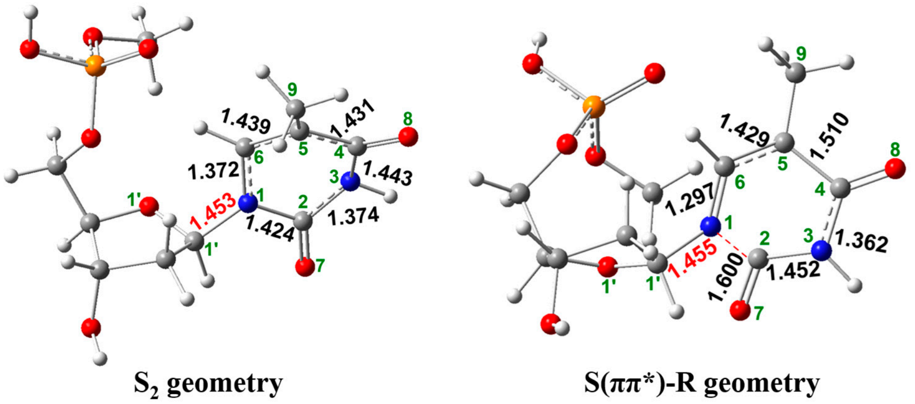


**Figure S5. Comparison between $S_2$ geometry and S(ππ*)-R geometry.**

To explain why $S_0 \rightarrow S(\pi\pi^*)$ transition is chosen at the first step, the spectrum of neutral 5′-dTMPH is shown in Figure S3, and the main parameters for the vertical excitations are shown in Table S2. $S_0 \rightarrow S_2$, $S_0 \rightarrow S(\pi\pi^*)$, and $S_0 \rightarrow S_5$ transitions based on the $S_0$ geometry are shown in Figure S4, because their oscillator strengths are greater than 0.01. The first five low-lying excitations are examined, given that absorption spectra are usually dominated by the first few excited states.

Firstly, $S_0 \rightarrow S_1$ and $S_0 \rightarrow S_3$ transitions are excluded due to the very small oscillator strengths. Secondly, for $S_0 \rightarrow S_5$ transition, the dominant transition is $p_{(N3,O7\&O8\ of\ T)} \rightarrow \pi^*_{(T)}$, where $\pi^*_{(T)}$ has MO coefficients on the N1 and C1' atoms but the of $p_{(N3,O7\&O8\ of\ T)}$ has no MO coefficients on the N1 or C1' atom. That means upon electronic excitation, there is only one factor (increase antibonding nature) affecting the C1'-N1 bond in the $S_0 \rightarrow S_5$ transition, resulting in a little effect on the focused C1'-N1 bond. Thirdly, for $S_0 \rightarrow S_2$ and $S_0 \rightarrow S(\pi\pi^*)$ transitions, both of them have relatively large oscillator strengths, and the dominant transition features $\pi_{(T)} \rightarrow \pi^*_{(T)}$ where $\pi_{(T)}$ has MO coefficient on the N1 atom and $\pi^*_{(T)}$ has MO coefficients on the N1 and C1' atoms. It means that in $S_0 \rightarrow S_2$ and $S_0 \rightarrow S(\pi\pi^*)$ transitions, two factors (decrease bonding and increase antibonding natures) are included to impact the C1'-N1 bond, resulting in the large effect on the focused C1'-N1 bond. As discussed above, the transition involving one factor affecting the C1'-N1 bond is weaker than those involving two factors. By the same reason in subsection 1.1, only the more important transitions involving two factors, that is $S_0 \rightarrow S_2$ and $S_0 \rightarrow S(\pi\pi^*)$ transitions, are chosen at the first step in this study.

To verify why $S_0 \rightarrow S(\pi\pi^*)$ transition is selected rather than $S_0 \rightarrow S_2$ transition, the $S_2$ geometry and S(ππ*)-R geometry are obtained by geometrical optimizations using TD-M06-2X/6-31G(d) method, as shown in Figure S5. It was found that these two geometries have similar energies and no special difference on the thermal stability as intermediate structures. However, Alexandrova et al. [S1] studied the nonadiabatic processes of small DNA fragments under the effect of UV light, through the semiclassical

nonadiabatic dynamics and the fewest switches surface hopping algorithm. The results for the thymine 4H'-nucleoside showed that, most trajectories (76.2%) proceeded via elongating the N1-C2 bond with the bending of thymine ring, whereas a minority (18.98%) involved the bending of thymine ring alone or tiny N2-C3 bond elongation. Based on our calculations in Figure S5, the S(ππ*)-R geometry refers to the N1-C2 bond elongation together with the bending of thymine ring, whereas the $S_2$ geometry only involves the bending of thymine ring. These results indicate that the S(ππ*)-R geometry might correspond to the hopping point [S1] with the elongated N1-C2 bond and the thymine ring bending which exists in the majority of trajectories. Besides, from the results of the above-mentioned study [S1], the structure with the N1-C2 bond cleavage could be formed staring from the hopping point. Accordingly, there might exist a path from the S(ππ*)-R geometry to the N1-C2 bond cleavage structure, which could compete with the glycosidic bond cleavage. To confirm it, the path of the N1-C2 bond cleavage will be examined in our subsequent study. That means the S(ππ*)-R geometry plays a crucial role in discussing the studied mechanism. Conversely, it can be inferred that the corresponding $S_0 \rightarrow S(\pi\pi^*)$ transition is very important. At the first step, therefore, this study only concentrates on the $S_0 \rightarrow S(\pi\pi^*)$ transition, although $S_0 \rightarrow S_2$ transition has a larger oscillator strength and has the excitation nature ππ*. $S_0 \rightarrow S_2$ transition and the other possible transitions will be examined in subsequent studies.

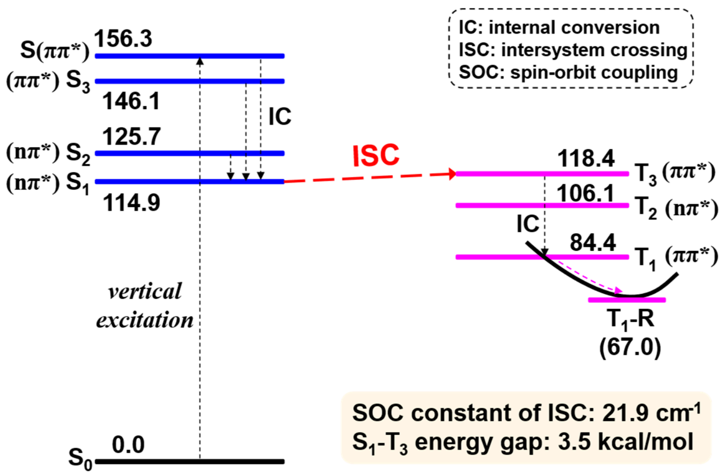


**Figure S6. ISC from singlet to triplet states with the excitation energies (kcal/mol), the calculated SOC constant ($cm^{-1}$) at B3LYP-D3BJ/6-31G(d) level using ORCA 5.0.4, and the $S_1$-$T_3$ energy gap (kcal/mol) for ISC.**

Due to the zero oscillator strength for the $S_0 \rightarrow S_1$ transition, $S_1$ should be from the IC involving the higher singlet ESs. To better understand this process, Figure S6 shows the energy profile including the $S_1$-S(ππ*) and $T_1$-$T_3$ states, which corresponds to the inset at the bottom right corner of Figure 6a in the main text.

As shown in Figure S6, upon excitation, the electron may be excited to the $S_2$-S(ππ*) states, and then go to the $S_1$ state via the IC. After that, the ISC may take place from $S_1$ to $T_3$ states. The high SOC constant of 21.9 $cm^{-1}$ and the low $S_1$-$T_3$ energy gap of 3.5 kcal/mol, indicate that this ISC can occur efficiently from $S_1$ to $T_3$ states. Apart from the energy gap and SOC constant mentioned above, it is also necessary to examine the IC and ISC rates which are essential to understand the branching ratio of these two reaction channels. If the IC rates are greatly higher than the ISC rates, the ISC will be unfeasible. To gain a thorough understanding of the studied mechanism, the IC and ISC rates will be calculated and analyzed in our future study.

## 2. References

S1. Alexandrova, A.N.; Tully, J.C.; Granucci, G. Photochemistry of DNA Fragments via Semiclassical Nonadiabatic Dynamics. *J. Phys. Chem. B* **2010**, *114*, 12116−12128.

# 3. Coordinates

## $D_0$

| | | | |
|---|---|---|---|
| O | -1.62603500 | -1.94189900 | 1.40782700 |
| P | -2.25783300 | -1.77305800 | -0.03742600 |
| O | -3.81177400 | -2.03093800 | 0.32765700 |
| O | -1.76312600 | -2.60695800 | -1.14049800 |
| O | -2.19786200 | -0.20801400 | -0.36649600 |
| C | -2.66131900 | 0.73764100 | 0.60090800 |
| C | -1.77022400 | 1.96482500 | 0.55143800 |
| O | -0.46426200 | 1.59209200 | 0.92716200 |
| C | -1.66125800 | 2.64588100 | -0.83097700 |
| O | -1.63806900 | 4.06055600 | -0.69963700 |
| C | -0.35277100 | 2.08882700 | -1.37368500 |
| C | 0.49938200 | 1.93540500 | -0.10977600 |
| N | 1.53100600 | 0.96814000 | -0.16946200 |
| C | 2.69522900 | 1.25701700 | 0.52750100 |
| O | 2.87463000 | 2.32339800 | 1.11911900 |
| N | 3.63619600 | 0.27391300 | 0.48428100 |
| C | 3.52198500 | -1.06086500 | -0.07008800 |
| O | 4.49181300 | -1.81995400 | 0.12688800 |
| C | 2.31645800 | -1.28333300 | -0.74737900 |
| C | 2.08616900 | -2.61981700 | -1.39234000 |
| C | 1.35287200 | -0.26079800 | -0.90357400 |
| H | 0.31055800 | -0.53860900 | -1.04729000 |
| H | 4.47577100 | 0.44927000 | 1.01706000 |
| H | 1.04166400 | -2.94642800 | -1.28348500 |
| H | 2.74748100 | -3.36270800 | -0.93745100 |
| H | 2.29567400 | -2.60665400 | -2.47211700 |
| H | 0.97225800 | 2.87635300 | 0.18703500 |
| H | 0.10558800 | 2.76587400 | -2.09866700 |
| H | -0.50780600 | 1.11734200 | -1.84364000 |
| H | -2.52305400 | 2.42353400 | -1.46623800 |
| H | -2.18192400 | 2.69962400 | 1.26058200 |
| H | -2.61187000 | 0.30185000 | 1.60449700 |
| H | -3.70232600 | 0.99407600 | 0.37158500 |
| H | -4.14805300 | -2.71023300 | -0.27597400 |
| C | -0.20639900 | -1.70900300 | 1.58603500 |
| H | 0.00797700 | -0.64168300 | 1.49367200 |
| H | 0.01935600 | -2.06548300 | 2.59079800 |
| H | 0.37194800 | -2.26570800 | 0.84632600 |
| H | -0.81941100 | 4.29014000 | -0.23359000 |

## $D_0$-TS

| | | | |
|---|---|---|---|
| O | -2.43550500 | 1.50935900 | -1.54339200 |
| P | -2.54129100 | 1.55045500 | 0.04043700 |
| O | -4.14901300 | 1.52799900 | 0.19531800 |
| O | -1.88981900 | 2.64311400 | 0.77552700 |
| O | -2.06330100 | 0.10603700 | 0.52972800 |
| C | -2.67517800 | -1.05856400 | -0.03636900 |
| C | -1.67852200 | -2.19937700 | -0.03946000 |
| O | -0.62493700 | -1.91356400 | -0.94502600 |
| C | -1.01806900 | -2.51095900 | 1.31858800 |
| O | -0.86333200 | -3.91297700 | 1.48683800 |
| C | 0.32929400 | -1.80142200 | 1.18922500 |
| C | 0.61215300 | -1.87024600 | -0.27698500 |
| N | 1.39281100 | -0.22926500 | -0.80532300 |
| C | 2.66328200 | -0.59655500 | -1.24412400 |
| O | 2.85514200 | -1.48381000 | -2.07420100 |
| N | 3.72799400 | 0.07093300 | -0.67558600 |
| C | 3.71321100 | 1.00885300 | 0.38473200 |
| O | 4.79289800 | 1.42975600 | 0.82827500 |
| C | 2.39708500 | 1.36895000 | 0.77793900 |
| C | 2.21587100 | 2.42361900 | 1.82824600 |
| C | 1.32474800 | 0.79873200 | 0.10000500 |
| H | 0.32569400 | 1.19104400 | 0.28744300 |
| H | 4.64301700 | -0.19589900 | -1.01473800 |
| H | 1.18415000 | 2.79247400 | 1.82902400 |
| H | 2.89435400 | 3.26675400 | 1.65648600 |
| H | 2.43867900 | 2.04534900 | 2.83541700 |
| H | 1.32349600 | -2.58426500 | -0.68464500 |
| H | 1.09652900 | -2.26536400 | 1.81415200 |
| H | 0.24115500 | -0.73896300 | 1.48582800 |
| H | -1.62615200 | -2.16851600 | 2.16139200 |
| H | -2.21517800 | -3.09819000 | -0.37688100 |
| H | -2.98901100 | -0.85249700 | -1.06570800 |
| H | -3.55746100 | -1.31520600 | 0.56137300 |
| H | -4.39370900 | 2.19633000 | 0.85307000 |
| C | -1.12866400 | 1.25460500 | -2.11501100 |
| H | -0.75626900 | 0.26889700 | -1.81816100 |
| H | -1.26655100 | 1.30397800 | -3.19454000 |
| H | -0.42345200 | 2.02249600 | -1.78893400 |
| H | -0.15389100 | -4.17004900 | 0.87400300 |

## $D_1$-R

| | | | |
|---|---|---|---|
| O | 1.28202800 | -1.77060900 | -1.39758400 |
| P | 2.05112700 | -1.62084200 | -0.02366100 |
| O | 2.93359100 | -2.97210100 | 0.09755000 |
| O | 1.26242400 | -1.40157700 | 1.20556200 |
| O | 3.23651700 | -0.60157700 | -0.34620700 |
| C | 3.05279800 | 0.60762400 | -1.08791800 |
| C | 1.99236200 | 1.52010800 | -0.51110500 |
| O | 0.74090100 | 0.92955600 | -0.70027900 |
| C | 2.09021800 | 1.81646500 | 0.99736000 |
| O | 2.82460800 | 3.02318600 | 1.14770600 |
| C | 0.61344900 | 1.88391200 | 1.43264200 |
| C | -0.18331800 | 1.69154200 | 0.12915100 |
| N | -1.44265800 | 1.06704400 | 0.21219700 |
| C | -2.31827400 | 1.25679300 | -0.99883200 |
| O | -2.16963200 | 2.29518700 | -1.69081500 |
| N | -3.67755200 | 0.90953000 | -0.55855500 |
| C | -3.92603600 | -0.28343500 | 0.06098000 |
| O | -5.03402700 | -0.82418700 | 0.07091400 |
| C | -2.76560000 | -0.83134700 | 0.79343400 |
| C | -2.96748400 | -2.12547100 | 1.52583300 |
| C | -1.62524600 | -0.10437100 | 0.88894600 |
| H | -0.78978600 | -0.47280200 | 1.47761500 |
| H | -4.40339800 | 1.24194600 | -1.17998500 |
| H | -3.78382700 | -2.05431500 | 2.25502400 |
| H | -2.05169100 | -2.41896300 | 2.05167400 |
| H | -3.24335700 | -2.93484100 | 0.83885900 |
| H | -0.35306000 | 2.64178700 | -0.38786500 |
| H | 0.38219600 | 2.84745900 | 1.89602200 |
| H | 0.39105300 | 1.08490400 | 2.14166500 |
| H | 2.60255800 | 0.99093000 | 1.50692100 |
| H | 2.05940000 | 2.49096300 | -1.03487300 |
| H | 2.78574800 | 0.35400400 | -2.11857200 |
| H | 4.02576700 | 1.10404300 | -1.06758100 |
| H | 2.68088500 | -3.40326200 | 0.92761200 |
| C | -0.16333100 | -1.79573300 | -1.44824600 |

| | | | |
|---|---|---|---|
| H | -0.55266100 | -0.78352000 | -1.58643000 |
| H | -0.41742900 | -2.43132300 | -2.29738000 |
| H | -0.57355000 | -2.21284600 | -0.52696600 |
| H | 2.90719500 | 3.19288500 | 2.09542900 |

## D$_1$-TS

| | | | |
|---|---|---|---|
| O | 0.05297000 | 2.14477300 | 1.45655500 |
| P | 0.61121100 | 2.37536900 | -0.00919000 |
| O | 1.67689900 | 3.55149800 | 0.33610300 |
| O | -0.26971100 | 2.75562200 | -1.12197800 |
| O | 1.55669700 | 1.11201500 | -0.30740500 |
| C | 2.60183400 | 0.82253200 | 0.61912600 |
| C | 2.93212500 | -0.65433500 | 0.56669000 |
| O | 1.86031700 | -1.43245200 | 1.09615600 |
| C | 3.18743900 | -1.23905200 | -0.83237500 |
| O | 4.10958200 | -2.31589400 | -0.74928000 |
| C | 1.79043200 | -1.71203600 | -1.24588300 |
| C | 1.15694700 | -2.03343600 | 0.07117000 |
| N | -1.06352600 | -1.63788500 | -0.70395300 |
| C | -1.76979500 | -2.19914500 | 0.31983900 |
| O | -1.54053900 | -3.33184600 | 0.75942400 |
| N | -2.78718000 | -1.42474600 | 0.92757400 |
| C | -3.48159400 | -0.45092300 | 0.14090500 |
| O | -4.66862400 | -0.19222300 | 0.41185300 |
| C | -2.67770600 | 0.14186600 | -0.86768400 |
| C | -3.26588600 | 1.17248300 | -1.77392000 |
| C | -1.31517400 | -0.31685500 | -1.04068500 |
| H | -0.77971700 | 0.04787800 | -1.91594100 |
| H | -3.41209200 | -1.98301300 | 1.49716000 |
| H | -3.36695100 | 0.78720800 | -2.79911400 |
| H | -2.60655200 | 2.04783300 | -1.82385800 |
| H | -4.25567000 | 1.46598000 | -1.41549700 |
| H | 0.64793900 | -2.94246000 | 0.35923700 |
| H | 1.84057600 | -2.56628900 | -1.92774500 |
| H | 1.23786300 | -0.90787300 | -1.74482500 |
| H | 3.62794900 | -0.50740500 | -1.51416200 |
| H | 3.81125200 | -0.82411400 | 1.20006600 |
| H | 2.28929400 | 1.08117300 | 1.63723900 |
| H | 3.48589600 | 1.41775700 | 0.36015600 |
| H | 1.54059100 | 4.25565200 | -0.31505400 |

| | | | |
|---|---|---|---|
| C | -0.72551900 | 0.97543600 | 1.79823900 |
| H | -0.21939000 | 0.06718900 | 1.46358200 |
| H | -0.80802200 | 0.99451300 | 2.88554800 |
| H | -1.70913400 | 1.03682400 | 1.33420600 |
| H | 3.64359600 | -3.03413100 | -0.29243900 |

## D-P

| | | | |
|---|---|---|---|
| O | -2.05175500 | -2.01896000 | 0.99935100 |
| P | -2.60950700 | -1.84338500 | -0.46457000 |
| O | -4.15419500 | -2.26001800 | -0.22244100 |
| O | -1.98520800 | -2.55613600 | -1.58756800 |
| O | -2.65826800 | -0.25882300 | -0.71256800 |
| C | -3.45120400 | 0.57201700 | 0.13777700 |
| C | -2.73875600 | 1.88857200 | 0.39085000 |
| O | -1.74236000 | 1.71134000 | 1.39589000 |
| C | -1.99582400 | 2.47671500 | -0.82407900 |
| O | -1.99515300 | 3.89438400 | -0.75571400 |
| C | -0.58408500 | 1.91341400 | -0.63954400 |
| C | -0.48391900 | 1.91260500 | 0.85466500 |
| N | 1.88488100 | -0.04408300 | -0.13407000 |
| C | 2.36592700 | -0.18158800 | 1.12434500 |
| O | 1.66454100 | -0.20082200 | 2.14999100 |
| N | 3.75310400 | -0.30435500 | 1.27572600 |
| C | 4.71765300 | -0.29548100 | 0.27185200 |
| O | 5.91436200 | -0.40843000 | 0.54740300 |
| C | 4.15150600 | -0.14426500 | -1.04377400 |
| C | 5.08420000 | -0.12072700 | -2.21567500 |
| C | 2.78549300 | -0.03370400 | -1.13422700 |
| H | 2.34862800 | 0.07824600 | -2.13110500 |
| H | 4.08865900 | -0.40488400 | 2.22525900 |
| H | 4.52804700 | -0.00584100 | -3.15240100 |
| H | 5.67528900 | -1.04256600 | -2.27717400 |
| H | 5.80558400 | 0.70215300 | -2.13981500 |
| H | 0.31457400 | 1.44784600 | 1.42522900 |
| H | 0.16696400 | 2.54136500 | -1.12756900 |
| H | -0.48539500 | 0.89766600 | -1.04302800 |
| H | -2.46706700 | 2.20246500 | -1.77076600 |
| H | -3.47644800 | 2.61302400 | 0.75748000 |
| H | -3.63777500 | 0.07738600 | 1.09769300 |
| H | -4.40961900 | 0.74944300 | -0.36337400 |

| | | | |
|---|---|---|---|
| H | -4.41094400 | -2.86055300 | -0.93810700 |
| C | -0.77440300 | -1.40032600 | 1.34564800 |
| H | -0.20712000 | -1.08450000 | 0.46484000 |
| H | -0.98042500 | -0.53951800 | 1.97796700 |
| H | -0.20062500 | -2.14296400 | 1.89520700 |
| H | -1.37085600 | 4.11739800 | -0.04515400 |

## S$_0$

| | | | |
|---|---|---|---|
| O | -2.19435500 | -1.84047400 | 1.34458200 |
| P | -2.41700000 | -1.54581800 | -0.20745300 |
| O | -3.99490300 | -1.75007600 | -0.35252600 |
| O | -1.55712200 | -2.29630900 | -1.13409700 |
| O | -2.25090800 | 0.03022900 | -0.37904900 |
| C | -2.62441500 | 0.96719700 | 0.63687600 |
| C | -1.58154900 | 2.06582500 | 0.63971800 |
| O | -0.32582100 | 1.48539900 | 0.97077400 |
| C | -1.40362800 | 2.76950700 | -0.71931500 |
| O | -1.20159500 | 4.13827200 | -0.42406100 |
| C | -0.14283200 | 2.11364000 | -1.27394800 |
| C | 0.65163800 | 1.86499900 | 0.00656300 |
| N | 1.65272300 | 0.82364100 | -0.07949200 |
| C | 2.94696000 | 1.12525600 | 0.34253700 |
| O | 3.29961000 | 2.22807300 | 0.70671100 |
| N | 3.80690200 | 0.04753700 | 0.29422000 |
| C | 3.53501300 | -1.27133900 | -0.08874700 |
| O | 4.40835000 | -2.11420700 | -0.06448700 |
| C | 2.14707500 | -1.49968200 | -0.49747100 |
| C | 1.76504800 | -2.88415300 | -0.92318000 |
| C | 1.29416400 | -0.45933700 | -0.46654200 |
| H | 0.25263100 | -0.58296000 | -0.74867100 |
| H | 4.75717900 | 0.24430700 | 0.58764500 |
| H | 0.70754300 | -2.93031800 | -1.19416900 |
| H | 1.96705900 | -3.59847500 | -0.11782800 |
| H | 2.37277900 | -3.19965100 | -1.77700100 |
| H | 1.17398500 | 2.76543900 | 0.33373600 |
| H | 0.39784400 | 2.75428200 | -1.97565900 |
| H | -0.39416000 | 1.17152700 | -1.76838700 |
| H | -2.27709100 | 2.62398700 | -1.36828000 |
| H | -1.85545400 | 2.82243000 | 1.38490500 |
| H | -2.65393700 | 0.47318500 | 1.61221800 |

| | | | |
|---|---|---|---|
| H | -3.61610600 | 1.36688300 | 0.40310900 |
| H | -4.21676400 | -2.65286800 | -0.62993600 |
| C | -0.83840100 | -1.83977800 | 1.82722700 |
| H | -0.43723300 | -0.82124000 | 1.81117700 |
| H | -0.88005300 | -2.21307900 | 2.84940300 |
| H | -0.22061400 | -2.49776000 | 1.21005000 |
| H | -1.00046300 | 4.60328000 | -1.24868300 |

## S(ππ*)-R

| | | | |
|---|---|---|---|
| O | 1.19399000 | 1.93407000 | 1.49277300 |
| P | 1.26436700 | 2.11283800 | -0.08202000 |
| O | 2.46371500 | 3.16577700 | -0.21976000 |
| O | -0.00651200 | 2.43298400 | -0.75455000 |
| O | 1.95287100 | 0.81029200 | -0.70213200 |
| C | 2.97711300 | 0.12154800 | 0.02663100 |
| C | 2.51469700 | -1.30715000 | 0.28023000 |
| O | 1.21713600 | -1.26315000 | 0.85480900 |
| C | 2.39931400 | -2.19551300 | -0.96306600 |
| O | 2.63015900 | -3.51659600 | -0.51849900 |
| C | 0.94816500 | -1.97968500 | -1.38056600 |
| C | 0.25140400 | -1.81291200 | -0.02885000 |
| N | -0.92917400 | -0.96187400 | -0.03131100 |
| C | -1.96351500 | -1.02094400 | 1.18768000 |
| O | -1.56889400 | -1.62385700 | 2.18453400 |
| N | -3.23753200 | -1.31500000 | 0.55580300 |
| C | -3.68840300 | -0.31447200 | -0.25004300 |
| O | -4.83620500 | -0.12735600 | -0.60316400 |
| C | -2.55890700 | 0.53526400 | -0.78164800 |
| C | -2.88414700 | 1.81648000 | -1.44794700 |
| C | -1.26394700 | -0.05785100 | -0.89859700 |
| H | -0.59923600 | 0.23928500 | -1.70462600 |
| H | -3.90260400 | -1.91078600 | 1.03391000 |
| H | -2.68404000 | 2.64862000 | -0.75865800 |
| H | -3.93996800 | 1.83448300 | -1.72476800 |
| H | -2.24093100 | 1.99284200 | -2.31447200 |
| H | -0.08671900 | -2.76588500 | 0.38841500 |
| H | 0.53784400 | -2.81338600 | -1.95648500 |
| H | 0.88476200 | -1.06892700 | -1.97859300 |
| H | 3.11322600 | -1.90368000 | -1.74512700 |
| H | 3.20327400 | -1.78614300 | 0.98537900 |
| H | 3.15419800 | 0.61768500 | 0.98410100 |
| H | 3.89503900 | 0.15005300 | -0.56710700 |
| H | 2.24432300 | 4.02547500 | 0.17248300 |
| C | 0.02598600 | 1.36552300 | 2.13048900 |
| H | 0.17724700 | 0.29658300 | 2.27914800 |
| H | -0.07679800 | 1.88050900 | 3.08504500 |
| H | -0.86327300 | 1.53218000 | 1.51786500 |
| H | 2.46846300 | -4.12240800 | -1.25560200 |

## S(ππ*)-TS

| | | | |
|---|---|---|---|
| O | -0.59760600 | -2.31533300 | 1.50158000 |
| P | -0.80340600 | -2.28158200 | -0.06995600 |
| O | -1.89656200 | -3.43501400 | -0.26057100 |
| O | 0.38837600 | -2.39216800 | -0.92844400 |
| O | -1.60806100 | -0.93003200 | -0.36203900 |
| C | -2.68830700 | -0.51344400 | 0.48093800 |
| C | -2.75349400 | 1.00095600 | 0.43876200 |
| O | -1.52856900 | 1.55183700 | 0.91639700 |
| C | -2.95344100 | 1.60034800 | -0.95582300 |
| O | -3.64109100 | 2.81749200 | -0.76244400 |
| C | -1.50475800 | 1.81078000 | -1.42469900 |
| C | -0.76260600 | 2.02854200 | -0.12573100 |
| N | 0.85359800 | 1.12232000 | 0.01745400 |
| C | 1.77246900 | 1.33218400 | 1.09950500 |
| O | 1.39544100 | 1.51061900 | 2.25520900 |
| N | 3.06480900 | 1.68118300 | 0.60814200 |
| C | 3.63704300 | 0.73203300 | -0.21945400 |
| O | 4.83345400 | 0.60955100 | -0.39875400 |
| C | 2.62961000 | -0.09839100 | -0.94687400 |
| C | 3.09105100 | -1.21472900 | -1.80068200 |
| C | 1.28699300 | 0.36710300 | -0.95100500 |
| H | 0.63106200 | 0.14288700 | -1.78892500 |
| H | 3.68905500 | 2.16890000 | 1.24144200 |
| H | 2.85063200 | -2.16896300 | -1.31324500 |
| H | 4.16747300 | -1.14837400 | -1.96337500 |
| H | 2.55292800 | -1.22773800 | -2.75492300 |
| H | -0.37342700 | 3.02169300 | 0.08735200 |
| H | -1.40218100 | 2.65799600 | -2.10892700 |
| H | -1.18306000 | 0.90374100 | -1.94377600 |
| H | -3.51215500 | 0.92201300 | -1.61544600 |
| H | -3.55727400 | 1.34207300 | 1.09894000 |
| H | -2.50984600 | -0.84502800 | 1.50811400 |
| H | -3.62031200 | -0.95883300 | 0.11676500 |
| H | -1.53478400 | -4.16657500 | -0.78435200 |
| C | 0.24380000 | -1.29529500 | 2.08645700 |
| H | -0.22903400 | -0.31248000 | 2.01396900 |
| H | 0.37568500 | -1.57266900 | 3.13039000 |
| H | 1.21349500 | -1.27593000 | 1.58093200 |
| H | -3.68924700 | 3.28036500 | -1.61059800 |

## S(ππ*)-TS’

| | | | |
|---|---|---|---|
| C | -2.54703500 | -1.01662200 | 0.69313200 |
| O | -1.75060700 | 0.00978900 | 1.31337500 |
| C | -3.40420700 | -0.26089000 | -0.31874000 |
| C | -2.43760900 | 0.84257700 | -0.78493000 |
| C | -1.54878900 | 1.01637500 | 0.42739100 |
| N | 0.18312300 | 0.66958900 | -0.16163500 |
| C | 0.61548200 | -0.64919100 | -0.49453600 |
| O | -0.09517200 | -1.40152900 | -1.16465700 |
| N | 1.75022800 | -1.03777700 | 0.26386800 |
| C | 2.84188700 | -0.19475500 | 0.13761700 |
| O | 3.99404000 | -0.51902500 | 0.34778000 |
| C | 2.45915400 | 1.19508900 | -0.21122600 |
| C | 1.10647600 | 1.58396600 | -0.08353900 |
| H | 0.80126400 | 2.60427000 | 0.16159900 |
| H | 1.93709100 | -2.02760400 | 0.37758800 |
| H | -1.43710100 | 1.98453000 | 0.91001800 |
| H | -2.94028000 | 1.76974900 | -1.06977400 |
| H | -1.84472500 | 0.48539300 | -1.63547900 |
| H | -3.74048500 | -0.89901400 | -1.13756100 |
| H | -3.11037700 | -1.50257200 | 1.49076100 |
| H | -1.88392300 | -1.73137200 | 0.19528800 |
| H | -4.27846100 | 0.17473100 | 0.17413300 |
| H | 3.26530500 | 1.90602600 | -0.34521900 |

## $S_0$/S(ππ*)-CI

| | | | |
|---|---|---|---|
| C | -2.44041200 | -1.10899100 | 0.37763800 |
| O | -1.44150600 | -0.41630700 | 1.16437100 |
| C | -3.39775300 | -0.00223500 | -0.06042400 |

| | | | |
|---|---|---|---|
| C | -2.49126400 | 1.24254000 | -0.08455100 |
| C | -1.41290900 | 0.83874900 | 0.86165400 |
| N | 0.31473600 | 0.09576400 | -1.51817000 |
| C | 0.77175300 | -1.08959100 | -0.95605100 |
| O | 0.29248600 | -2.15293700 | -1.23786300 |
| N | 1.79397100 | -1.01438100 | -0.03601100 |
| C | 2.36666100 | 0.14047100 | 0.48179400 |
| O | 3.21313100 | 0.08468600 | 1.34233300 |
| C | 1.83517500 | 1.35091600 | -0.11193200 |
| C | 0.87203000 | 1.22358200 | -1.11203700 |
| H | 0.50927700 | 2.11638100 | -1.59472600 |
| H | 2.09594700 | -1.88348400 | 0.34963400 |
| H | -0.74691600 | 1.45723900 | 1.42433200 |
| H | -2.98806500 | 2.15438800 | 0.22794900 |
| H | -2.07663800 | 1.40778600 | -1.07446800 |
| H | -3.84146900 | -0.21257000 | -1.02361300 |
| H | -2.87741200 | -1.85172800 | 1.02509100 |
| H | -1.92626900 | -1.58426800 | -0.44183900 |
| H | -4.19341900 | 0.12337100 | 0.66453500 |
| H | 2.25217600 | 2.29651300 | 0.17027400 |

## S(ππ*)-P

| | | | |
|---|---|---|---|
| O | 0.62835300 | 2.83503900 | 0.66567700 |
| P | -0.02246700 | 1.80288000 | -0.36120400 |
| O | 0.02341900 | 2.65169600 | -1.71410300 |
| O | -1.33626100 | 1.24748000 | 0.00260000 |
| O | 1.09099800 | 0.67686100 | -0.54963200 |
| C | 2.42296200 | 1.06684800 | -0.92014100 |
| C | 3.37922200 | 0.00684800 | -0.41913900 |
| O | 3.30554300 | -0.02841700 | 1.00701700 |
| C | 3.07240200 | -1.41333600 | -0.91438800 |
| O | 4.32738800 | -2.02371200 | -1.14461000 |
| C | 2.30663600 | -2.03811100 | 0.25973600 |
| C | 2.82345000 | -1.24691300 | 1.41713500 |
| N | -4.24952900 | -0.31056900 | 0.78602700 |
| C | -4.14873200 | -0.54140500 | -0.59558500 |
| O | -5.02676900 | -0.22791200 | -1.36569300 |
| N | -2.99767700 | -1.18290200 | -1.06583000 |
| C | -1.86629300 | -1.46489100 | -0.33489900 |
| O | -0.86382000 | -1.94740800 | -0.83482400 |
| C | -1.99881100 | -1.14928100 | 1.09357600 |
| C | -0.81984000 | -1.35185700 | 1.96684600 |
| C | -3.22680700 | -0.60323700 | 1.53813100 |
| H | -3.33572500 | -0.39441300 | 2.60317800 |
| H | -2.95339000 | -1.33995500 | -2.06638600 |
| H | -0.00561700 | -0.69564000 | 1.62732300 |
| H | -0.44240100 | -2.37708400 | 1.88162800 |
| H | -1.04960700 | -1.13058400 | 3.01162700 |
| H | 2.42626800 | -1.24332400 | 2.42553300 |
| H | 2.52252100 | -3.10983900 | 0.34693400 |
| H | 1.22648200 | -1.92640300 | 0.09468500 |
| H | 2.46543000 | -1.39488500 | -1.82934500 |
| H | 4.39835000 | 0.27769900 | -0.71428600 |
| H | 2.66822100 | 2.03215900 | -0.46467900 |
| H | 2.47502800 | 1.15851600 | -2.00969600 |
| H | -0.87228700 | 2.81301700 | -2.05085900 |
| C | 0.92097200 | 2.33013700 | 1.97772700 |
| H | 1.71366800 | 1.57665500 | 1.92008500 |
| H | 1.25558500 | 3.18285100 | 2.56645700 |
| H | 0.01765400 | 1.90429900 | 2.42451600 |
| H | 4.17549300 | -2.95979500 | -1.33547700 |

## $T_1$-R

| | | | |
|---|---|---|---|
| O | 1.17037100 | -2.10289200 | -1.49158800 |
| P | 1.65177400 | -2.11716400 | 0.02989300 |
| O | 3.05799000 | -2.86902600 | -0.08949400 |
| O | 0.67732900 | -2.64707200 | 0.99529600 |
| O | 2.11015000 | -0.62925800 | 0.37753300 |
| C | 2.77796000 | 0.19757100 | -0.58416000 |
| C | 2.14931600 | 1.57772400 | -0.54456000 |
| O | 0.77277700 | 1.44803200 | -0.86597400 |
| C | 2.21965600 | 2.29528300 | 0.81503800 |
| O | 2.45761500 | 3.65884800 | 0.51924400 |
| C | 0.82718400 | 2.07003600 | 1.39680300 |
| C | -0.04144500 | 2.05011700 | 0.14041800 |
| N | -1.25954200 | 1.27318700 | 0.25813000 |
| C | -2.28652300 | 1.61416300 | -0.63787800 |
| O | -2.30877100 | 2.66571300 | -1.23875600 |
| N | -3.26460300 | 0.65066800 | -0.81062300 |
| C | -3.49440800 | -0.49402100 | -0.01594300 |
| O | -4.54995900 | -1.10760200 | -0.11885200 |
| C | -2.43787400 | -0.79029500 | 0.90624600 |
| C | -2.55619700 | -1.87918300 | 1.89807000 |
| C | -1.20580800 | 0.04433000 | 0.90607600 |
| H | -0.24427600 | -0.44892100 | 0.96404800 |
| H | -4.03734500 | 0.94027900 | -1.39796600 |
| H | -1.69546900 | -2.55582700 | 1.80206300 |
| H | -3.49113500 | -2.42467400 | 1.76537900 |
| H | -2.50713900 | -1.46601100 | 2.91577600 |
| H | -0.32871500 | 3.05213300 | -0.17982000 |
| H | 0.52722900 | 2.85908700 | 2.09163600 |
| H | 0.78634800 | 1.11029600 | 1.91560300 |
| H | 3.01207900 | 1.88173200 | 1.45294000 |
| H | 2.65034200 | 2.21177600 | -1.28654000 |
| H | 2.66196600 | -0.22596400 | -1.58590600 |
| H | 3.84110700 | 0.23966700 | -0.32902800 |
| H | 2.95662000 | -3.83381600 | -0.10056300 |
| C | -0.16766100 | -1.66221600 | -1.78653700 |
| H | -0.21533500 | -0.57181300 | -1.72148000 |
| H | -0.37805800 | -1.99150500 | -2.80314500 |
| H | -0.87700200 | -2.12149200 | -1.09082200 |
| H | 2.42598800 | 4.16052700 | 1.34617800 |

## $T_1$-TS

| | | | |
|---|---|---|---|
| O | -1.78746500 | -2.24965500 | 1.17546000 |
| P | -1.68532900 | -2.00693100 | -0.39249000 |
| O | -3.13129000 | -2.52167800 | -0.85949300 |
| O | -0.54492100 | -2.58643200 | -1.11478400 |
| O | -1.78752600 | -0.41841500 | -0.57841200 |
| C | -2.81322900 | 0.29754900 | 0.11536700 |
| C | -2.32575100 | 1.70759500 | 0.35605800 |
| O | -1.19323100 | 1.68910600 | 1.22795400 |
| C | -1.87755400 | 2.46067200 | -0.90312100 |
| O | -2.29348800 | 3.79991800 | -0.72769000 |
| C | -0.34873700 | 2.31040200 | -0.87656700 |
| C | -0.05385800 | 1.95794500 | 0.54501300 |
| N | 1.01669400 | 0.22095500 | 0.38391500 |
| C | 1.92626700 | 0.31629400 | 1.45896500 |
| O | 1.55389500 | 0.62638600 | 2.57146800 |
| N | 3.24870700 | 0.00282400 | 1.20075300 |

| | | | |
|---|---|---|---|
| C | 3.82962800 | -0.16535300 | -0.06393700 |
| O | 5.04650300 | -0.22909000 | -0.19366800 |
| C | 2.86769700 | -0.25623100 | -1.13264200 |
| C | 3.31047800 | -0.32268200 | -2.54428100 |
| C | 1.44246600 | -0.23429700 | -0.77997500 |
| H | 0.70403900 | -0.58479400 | -1.49447200 |
| H | 3.88188600 | 0.05085300 | 1.99062300 |
| H | 2.84513100 | -1.17907700 | -3.04842400 |
| H | 4.39681500 | -0.40529600 | -2.59869800 |
| H | 2.99068500 | 0.57454200 | -3.09231800 |
| H | 0.67894000 | 2.47785800 | 1.15146900 |
| H | 0.15193200 | 3.22869100 | -1.19859500 |
| H | -0.02057400 | 1.49684900 | -1.54440200 |
| H | -2.32037600 | 2.02272100 | -1.80737300 |
| H | -3.11664300 | 2.27800200 | 0.85313100 |
| H | -3.02710800 | -0.18444600 | 1.07447900 |
| H | -3.72372900 | 0.30267900 | -0.49381200 |
| H | -3.04484300 | -3.31752400 | -1.40676800 |
| C | -0.71294200 | -1.79516000 | 2.02103800 |
| H | -0.69819300 | -0.70307900 | 2.06826800 |
| H | -0.90726700 | -2.22097800 | 3.00431300 |
| H | 0.24294000 | -2.15976300 | 1.63599300 |
| H | -1.97811400 | 4.31873800 | -1.48093300 |
| N | -3.12433500 | -1.54418100 | 0.75908700 |
| C | -3.79068600 | -0.64079700 | -0.05558800 |
| O | -4.94701200 | -0.31872400 | 0.14442300 |
| C | -2.96693700 | -0.12696000 | -1.15572300 |
| C | -3.50848600 | 0.92366700 | -2.04619100 |
| C | -1.64335000 | -0.63477300 | -1.28119600 |
| H | -1.03972300 | -0.27122900 | -2.11227400 |
| H | -3.63543500 | -1.91298500 | 1.55412400 |
| H | -3.11247500 | 1.89057100 | -1.70858200 |
| H | -4.59795300 | 0.94541300 | -1.99838000 |
| H | -3.17277600 | 0.77935500 | -3.07726600 |
| H | 1.16755000 | -2.54738100 | 0.86032900 |
| H | 2.29692300 | -2.69709700 | -1.67527100 |
| H | 1.27197600 | -1.26539000 | -1.45600600 |
| H | 3.32982400 | -0.14065100 | -1.91877400 |
| H | 4.35963200 | -0.14110800 | 0.67005100 |
| H | 2.63249200 | 1.53890300 | 1.29920900 |
| H | 3.37169500 | 1.88853200 | -0.28910500 |
| H | 0.35395500 | 4.23629400 | -0.09730400 |
| C | -0.31903900 | 0.51879000 | 2.09356700 |
| H | 0.28862400 | -0.27409900 | 1.64796200 |
| H | -0.24791500 | 0.47408100 | 3.17897400 |
| H | -1.36546900 | 0.42750800 | 1.78653100 |
| H | 4.56187400 | -2.21038600 | -1.89882500 |

## $T_1$-P

| | | | |
|---|---|---|---|
| O | 0.18112000 | 1.81116700 | 1.70837300 |
| P | 0.29713500 | 2.10172400 | 0.14740500 |
| O | 0.98788400 | 3.54578900 | 0.15071200 |
| O | -0.94538700 | 1.99548000 | -0.63823000 |
| O | 1.45147800 | 1.13565200 | -0.37069100 |
| C | 2.74129600 | 1.18874300 | 0.26795000 |
| C | 3.34106900 | -0.20013800 | 0.27002900 |
| O | 2.55452100 | -1.03292900 | 1.11717500 |
| C | 3.38283200 | -0.87910000 | -1.10662100 |
| O | 4.60191600 | -1.59517000 | -1.15361100 |
| C | 2.15435100 | -1.79458600 | -1.06973400 |
| C | 2.02425400 | -2.07723100 | 0.39129300 |
| N | -1.07607300 | -1.48868400 | -0.47992500 |
| C | -1.79579600 | -1.94950900 | 0.63277800 |
| O | -1.28877200 | -2.66436100 | 1.47010200 |
